# Profiling the diversity of the village chicken faecal microbiota using 16S rRNA gene and metagenomic sequencing data to reveal patterns of gut microbiome signatures

**DOI:** 10.3389/fmicb.2024.1487595

**Published:** 2025-02-04

**Authors:** Mxolisi Nene, Nokuthula Winfred Kunene, Rian Pierneef, Khanyisile Hadebe

**Affiliations:** ^1^Department of Agriculture, University of Zululand, Kwa Dlangezwa, South Africa; ^2^Biotechnology Platform, Agricultural Research Council, Ondersterpoort, South Africa; ^3^Department of Biochemistry, Genetics and Microbiology, University of Pretoria, Pretoria, South Africa; ^4^Centre for Bioinformatics and Computational Biology, University of Pretoria, Pretoria, South Africa; ^5^DSI/NRF SARChI in Marine Microbiomics, Department of Biochemistry, Genetics and Microbiology, University of Pretoria, Pretoria, South Africa

**Keywords:** next generation sequencing, microbiome, communal production system, antimicrobial Resistance, gallus domesticus

## Abstract

**Introduction:**

The production environment of extensively raised village chickens necessitates their adaptability to low-resource systems. The gut microbiome plays a critical role in supporting this adaptability by influencing health and productivity. This study aimed to investigate the diversity and functional capacities of the faecal microbiome in village chickens from Limpopo and KwaZulu-Natal provinces of South Africa.

**Methods:**

Using a combination of 16S rRNA gene sequencing and shotgun metagenomic sequencing technologies, we analysed 98 16S rRNA and 72 metagenomic datasets. Taxonomic profiles and functional gene annotations were derived, focusing on microbial diversity, antibiotic resistance genes (ARGs), and potential zoonotic pathogens.

**Results:**

Taxonomic analysis showed that the predominant phyla in both provinces were Firmicutes, Bacteroidota, Proteobacteria, and Actinobacteria. At the genus level, *Escherichia* and *Shigella* were prevalent, with *Escherichia coli* and *Shigella dysenteriae* identified as major contributors to the gut microbiome. ARGs were identified, with *MarA*, *PmrF*, and *AcrE* detected in KwaZulu-Natal, and *cpxA*, *mdtG,* and *TolA* in Limpopo. These genes primarily mediate antibiotic efflux and alteration.

**Discussion:**

The detection of zoonotic bacteria such as *Escherichia coli* and S*treptococcus* spp. highlights potential health risks to humans through the food chain, emphasizing the importance of improved household hygiene practices. This study underscores the role of the gut microbiome in village chicken health and adaptability, linking microbial diversity to production efficiency in low-resource settings. Targeted interventions and further research are crucial for mitigating zoonotic risks and enhancing sustainability in village chicken farming.

## 1 Introduction

The chicken intestinal microbiome is composed of diverse communities, including prokaryotes, eukaryotes, and viruses, which have a significant impact on metabolism, production, and health (Keambou et al., 2014; Bahrndorff et al., 2016; McKenna et al., 2020). Understanding the structure and function of these communities is critical, particularly for village chickens that thrive in low-resource environments where the microbiome can influence resilience against pathogens and nutritional stress (Seidlerova et al., 2020). The gut microbiome has been shown to support the host’s adaptability, enabling animals to survive and maintain productivity under harsh environmental conditions similar to those found in traditional farming systems in a wide variety of agroecological zones (Suman et al., 2022).

Advances in next-generation sequencing (NGS) technologies, including 16S rRNA amplicon sequencing and shotgun metagenomics, have revolutionized our understanding of microbial diversity by enabling culture-independent assessments of microbiota (Hiergeist et al., 2015). These tools are good for the characterization of previously uncultivable microorganisms and assessment of microbial population dynamics in the cecum (Leggett et al., 2013; Cressman et al., 2010). Such an approach is extremely important for studies on village chickens since there is high evidence that the gut microbiome may play a significant role in health and productivity under conditions that contrast greatly with those in commercial production systems (Aruwa et al., 2021). Research indicates that the gut microbiota plays a vital role in the health and productivity of chickens. For instance, the composition of the gut microbiome can be influenced by various factors, including diet, environmental conditions, and farming practices. In commercial settings, practices such as antibiotic use and biosecurity measures can drastically alter the microbial landscape, often leading to reduced biodiversity and increased prevalence of pathogenic organisms (Muyyarikkandy et al., 2023; Shang et al., 2018). In contrast, village chickens, which are typically raised in low-input scavenging systems, may harbour a more diverse microbiome that could enhance their resilience to diseases and improve nutrient absorption (Morris, 2024). The presence of a rich and varied gut microbiota is crucial for optimal digestive efficiency and immune system development, which are particularly important in resource-limited environments (Saint-Martin et al., 2022; Tsega et al., 2019). While some studies have focused on the rise in microbiome research, studies on village chickens’ gut microbiota are still scant in South Africa, particularly those seeking to understand how regional environmental factors and traditional farming practices shape microbial communities (Mootane et al., 2024). Most microbiome studies have generally been conducted with commercial breeds in controlled environments, such as beef cattle (Clemmons et al., 2019), dairy cows (Andrews et al., 2019), pigs (Yang et al., 2014), poultry (Tan et al., 2019; Fidler et al., 2020), and sheep (Ellison et al., 2019; Patil et al., 2018). These studies are limited in unravelling the dynamics of the microbiome in natural, resource-limited settings typical for village chicken farming.

In light of the foregoing, this work study profiles the diversity and functional potential of the faecal microbiome in village chickens from two provinces: Limpopo and KwaZulu-Natal. These provinces were selected to represent the dissimilar agroecological regions under different environmental conditions and traditional poultry-keeping practices. Limpopo represents a dry and semi-arid climate, in contrast to the more humid and temperate environment of KwaZulu-Natal. The way these differences affect the intestinal microbiome could explain the role of microbiota in supporting chicken resilience to their native environments. Our hypothesis is that the gut microbiome plays a critical role in the adaptive capacity of village chickens, contributing to their ability to cope with the challenges imposed by low-input farming systems. This study attempts to provide an in-depth understanding of the taxonomic composition, microbial diversity, and functional potential of the faecal microbiome using both 16S rRNA gene and shotgun metagenomic sequencing. These insights will also reveal the presence of antibiotic resistance genes (ARGs) and its implications for public health and poultry management in rural South African communities.

## 2 Materials and methods

### 2.1 Faecal sample collection

**Figure 1:**
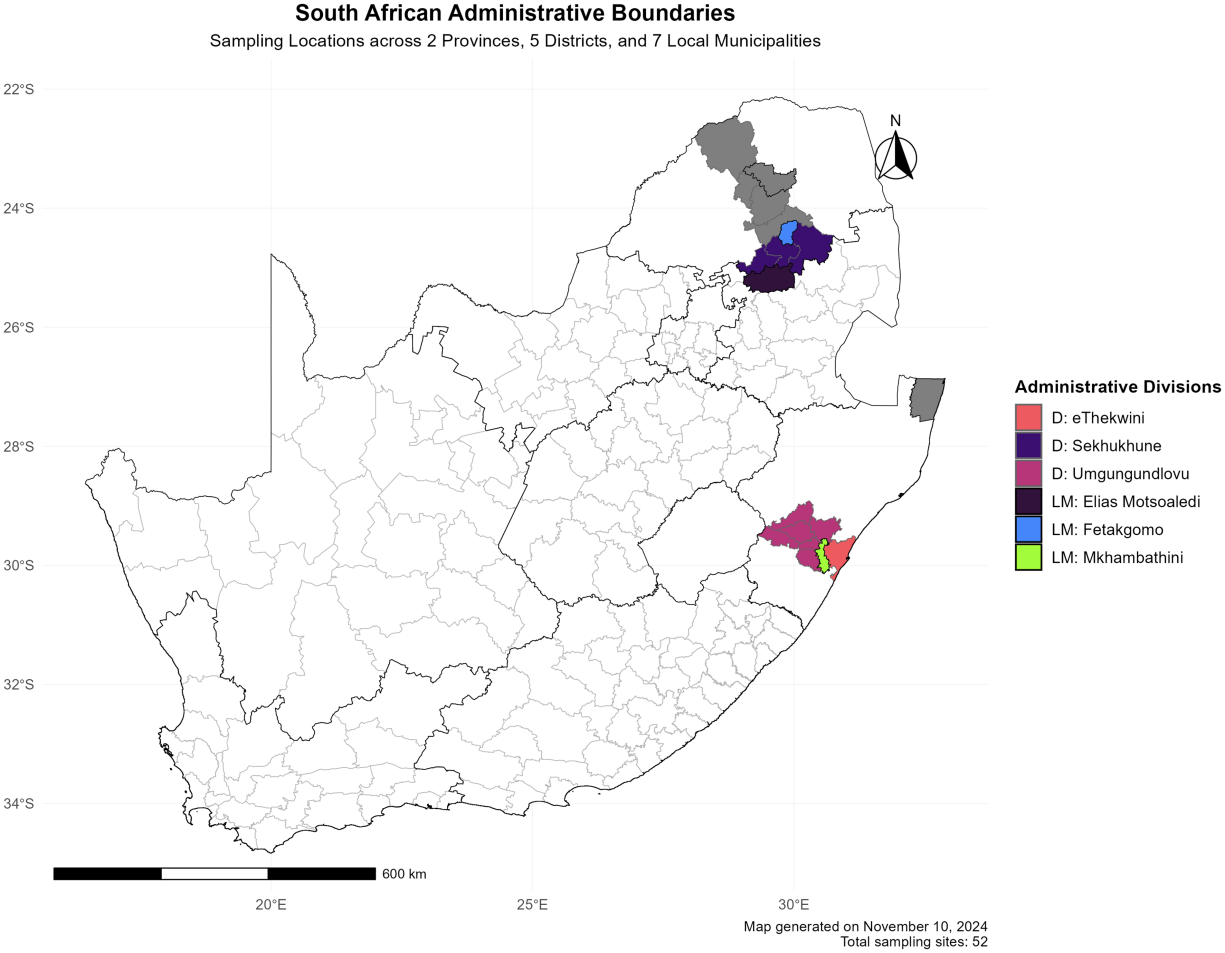
Spatial distribution of research locations in South Africa. The study encompasses two provinces (KwaZulu-Natal and Limpopo).

A total of 98 faecal samples were collected from village chickens in Limpopo and KwaZulu-Natal provinces. As shown in geographical map Figure 1; Three faecal samples were collected per household from the following district municipalities: eThekwini, uMgungundlovu, The Big 5 Hlabisa in KwaZulu-Natal and Sekhukhune, Capricon for Limpopo. KwaZulu-Natal local municipalities included eThekwini, uMkhambathini and Mkhanyakude; Elias Motsoaledi, Mole mole, and Fetakgomo local municipalities in Limpopo province. The chickens and homesteads were randomly selected based on different agroecological zones and environmental dynamics. The production system is characterised by extensive low input production. Non-invasive sampling techniques were used in order to do microbiome studies on free-range animals without catching and slaughtering them (Banks and Piggott, 2022).

Freshly voided faecal samples were collected using a sterile spatula and stored in 5 mL Eppendorf tubes (Eppendorf AC Barkhausenweg, Hamburg, Germany). Immediately after collection, these tubes were frozen on dry ice and stored at −80°C upon arrival at the Agricultural Research Council Biotechnology Platform until DNA extraction. All samples were collected by trained personnel from the Agricultural Research Council under ethical standards and guidelines (AEC 22/10).

### 2.2 DNA extraction, library preparation, and sequencing

Total faecal DNA was extracted using the ZymoBIOMICS DNA Miniprep Kit (Macherey-Nagel, Duren, Germany). To verify that sufficient DNA of good quality was obtained for 16S sequencing, quantification was performed on all extracted DNA samples using a Qubit dsDNA kit (Thermo Fisher Scientific, Massachusetts, USA). Library preparation of the V3 and V4 hypervariable regions of the 16S rRNA gene was carried out based on a 10 ng aliquot of DNA from each faecal sample.

The regions were subsequently amplified by polymerase chain reaction (PCR) using the following primer pair: forward 5′–TCG TCG GCA GCG TCA GAT GTG TAT AAG AGA CAG–3′ and reverse 5′–GTC TCG TGG GCT CGG AGA TGT GTA TAA GAG ACAG–3′ in a high-fidelity PCR buffer with enzyme mix under optimal conditions. First-round PCR products were then used as templates in a second round of amplicon enrichment with the following cycling parameters: 94°C for 3 min, followed by 24 cycles of 94°C for 5 s, 57°C for 90 s, and 72°C for 10 s, and a final extension at 72°C for 5 min. Indexed adapters were added to the ends of the 16S rRNA gene amplicons to create indexed libraries for subsequent next-generation sequencing (NGS) on the MiSeq platform (Gohl et al., 2016). DNA libraries were validated using an Agilent 2,100 Bioanalyzer (Agilent Technologies, Palo Alto, CA, USA) and quantified with a Qubit 2.0 Fluorometer. Then, libraries were multiplexed and loaded onto an Illumina MiSeq instrument (Illumina, San Diego, CA, USA) following the manufacturer’s instructions. Sequencing was done using a 2 × 300 paired-end setup, and image analysis with base calling was conducted with the MiSeq Control Software.

For metagenomics sequencing, DNBSEQ-G400 libraries were prepared using 500 ng of genomic DNA (gDNA) fragmented by Covaris E220 sonicator (Covaris, Brighton, UK). The sheared DNA was subjected to end repair and A-tailing as described in the MGI Easy Universal DNA Library Prep Set User Manual v1 (MGI Tech Co., Shenzhen, China). Adapter ligation was carried out as recommended by the MGIEasy DNA Adapters kit and purified using the accompanying DNA Clean Beads. PCR amplification was carried out on purified adapter-ligated DNA (95°C for 3 min, followed by 7 cycles of 98°C for 20 s, 60°C for 15 s, and 72°C for 30 s, with a final extension at 72°C for 10 min). Then, after quality control with the Qubit dsDNA HS Assay Kit (Thermo Fisher Scientific, Waltham, MD, USA), purified PCR products were denatured at 95°C for 3 min and then ligated to generate the single-strand circular DNA libraries. Barcode libraries were pooled with an equal amount of each one in order to generate DNA Nanoballs (DNB) and were then sequenced using the DNBSEQ-G400 technology of the MGI Tech Co. (Shenzhen, China) following the manufacturer’s instructions. Paired-end fastq files were generated for downstream analysis.

### 2.3 Data analysis

#### 2.3.1 16S rRNA gene data analysis

A total of 98 16S rRNA gene samples were used for analysis. The DADA2 pipeline (v1.16.0) implemented in R (v4.1.2; Callahan et al., 2016) was used to generate an amplicon sequence variant (ASV) table. The filterAndTrim function of the DADA2 pipeline was used to remove primers. The default settings were used for sequence filtering, trimming, error rate learning, dereplication, chimera removal, and amplicon sequence variant (ASV) inference. The SILVAngs (v138.1) database was utilised for taxonomic assignment (Gurevich et al., 2013) using the dada2-formatted training files for taxonomy and assignment up to the genus level (Callahan et al., 2016). Phyloseq (v1.24.2) was used to merge sample metadata, taxonomic assignment, and ASVs into a phyloseq object (McMurdie and Holmes, 2013). The programs phyloseq (v1.24.2) and ggplot2 (v3.3.5) were used for data handling and visualization. Alpha diversity indices Chao1, Shannon and the Observed ASVs were calculated. Beta diversity was estimated using the Bray–Curtis dissimilarity index and visualized using non-metric multidimensional scaling (NMDS). Differences in beta diversity were tested using permutational multivariate ANOVA (PERMANOVA).

#### 2.3.2 Metagenomic data analysis

A total of 72 metagenomic data sets were produced and analysed according to the procedure described by Chivian et al. (2021). Paired-end sequencing reads from fastq files underwent quality assessment with FastQC (Andrews et al., 2019) and quality improvement with Trimmomatic (Bolger et al., 2014). The quality filtered reads were taxonomically classified using Kaiju (Menzel et al., 2016) and the NCBI’s RefSeq (Tatusova et al., 2014). The quality filtered reads were assembled into contiguous fragments (contigs) using IDBA (Peng et al., 2011), MegaHit (Li et al., 2015), and MetaSpades (Nurk et al., 2017). The outputs were compared using QUAST (Gurevich et al., 2013) to determine the best assembly. The assembled contigs were clustered into bins using MaxBin2 (Wu et al., 2016) and MetaBAT (Kang et al., 2019). Thereafter CheckM was used to assess the bin Quality and medium-high quality Metagenome Assembles Genomes (MAG’s) were selected for downstream analysis (Parks et al., 2015). The MAGs were taxonomically classified and annotated using the Rapid Annotation Subsystem Technology (RAST) and Genome Taxonomic Database Toolkit (GTDB-TK; Chaumeil et al., 2022; Akhter et al., 2012). DRAM (Distilled and Refined Annotation of Metabolism; Shaffer et al., 2020) was used to profile MAGs for metabolic functionality known to impact ecosystem function across biomes. A set of Hidden Markov Models (HMMs) from the dbCAN2 CAZy collection was used to scan the MAGs (Chivian et al., 2023). The multiple sequence alignment generated by GTDB-TK was converted and edited with MEGA software version 7 (Tamura et al., 2013), and a circularized phylogenetic tree was generated and annotated on the Interactive Tree of Life (iTOL) version 5 (Letunic and Bork, 2021).

## 3 Results and discussion

### 3.1 16S rRNA gene microbial data

#### 3.1.1 Alpha diversity of the bacterial communities in the Limpopo and KwaZulu-Natal municipalities

**Figure 2:**
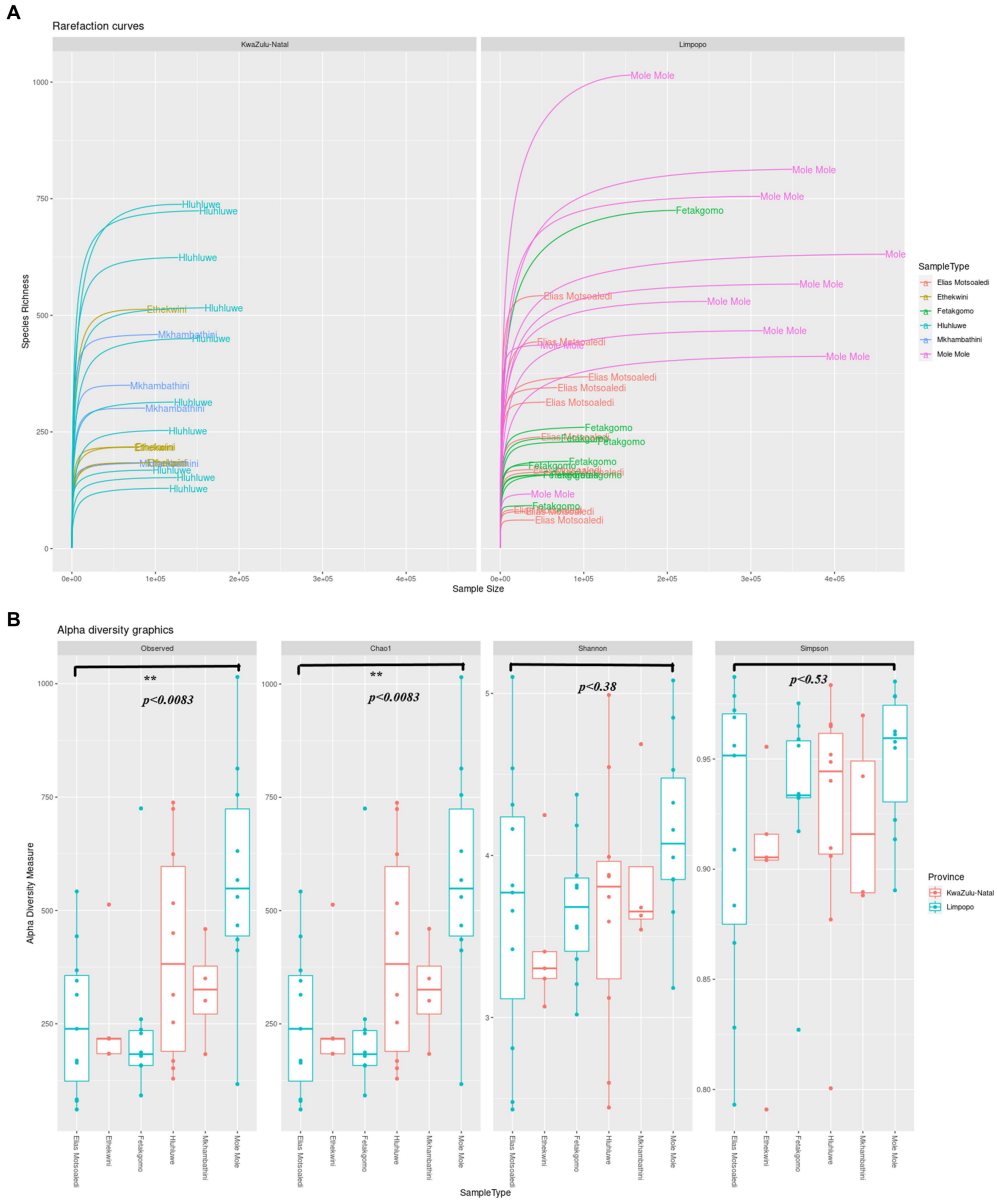
**(A)** The rarefaction curve of KwaZulu-Natal and Limpopo village chicken faecal samples. **(B)** Alpha diversity indexes, Observed ASVs, Chao1, Shannon and Simpson indices of KwaZulu-Natal and Limpopo villages.

A total of 17,456,214 sequence reads were obtained from all samples collected from KwaZulu-Natal and Limpopo provinces. The sequences were further run through the dada2 steps such as filtering, denoising, merging and removal of sequencing errors (Supplementary Figure 1). The percentage loss of input reads because of sequencing errors (chimeras) was (11.7%), and the non-chimeric reads accounted for (88.31%). As shown in Figure 2A, the rarefaction curves based on the Shannon index were almost flat, meaning the sequencing data was robust enough to reflect the bacterial communities in both KwaZulu-Natal and Limpopo provinces. The alpha diversity indices of the bacterial communities were significantly different between the provincial municipalities (Figure 2B; Supplementary Table 1). The mean Chao estimators were 351.54 and 348.31 in the KZN and Limpopo municipalities, respectively, indicating a higher abundance of bacterial communities in KZN than in Limpopo. Furthermore, the Shannon index, which reflects the evenness and diversity in the bacterial communities, ranged between 2.60 and 4.99 and 2.48 and 5.11 in KwaZulu-Natal and Limpopo, respectively. The KwaZulu-Natal sample X16S_KZN_NHL_H6 (4.99) and Limpopo sample X16S_Limp_EM_FC6 (5.11) had the highest Shannon indices. Together, these findings suggest that the diversity of bacterial communities in Limpopo was higher than that in the KwaZulu-Natal municipalities. In summary, the diversity of bacterial communities in Limpopo was higher than that in the KwaZulu-Natal and a further investigation of this phenomenon is necessary.

The analysis of bacterial communities in the KwaZulu-Natal (KZN) and Limpopo provinces reveals significant insights into microbial diversity, as indicated by the sequencing data processed through the dada2 pipeline. The total of 17,456,214 sequence reads generated from samples across these provinces underscores the robustness of the sequencing efforts, with a notable 88.31% of reads being non-chimeric, suggesting effective error correction and data integrity (Yin et al., 2019; Wan et al., 2018). The observed percentage loss of input reads (11.7%) due to sequencing errors is consistent with findings in other studies, where similar methodologies have been employed to assess microbial diversity (Wang et al., 2023; Du et al., 2019). The rarefaction curves based on the Shannon index indicate a stable representation of the bacterial communities, which is crucial for understanding the ecological dynamics within these regions. The Shannon index, a well-established metric for assessing biodiversity, reflects both the richness and evenness of species within a community (Yin et al., 2019). In this study, the mean Chao estimators of 351.54 for KZN and 348.31 for Limpopo suggest a slightly higher abundance of bacterial communities in KZN, although the Shannon indices indicate a higher diversity in Limpopo, with values ranging from 2.48 to 5.11 compared to KZN’s range of 2.60 to 4.99 (Zondo Sinenhlanhla et al., 2022). This discrepancy highlights the complex interactions within microbial ecosystems and suggests that environmental factors unique to Limpopo may be fostering greater diversity. Furthermore, the findings align with recent literature that emphasizes the role of environmental conditions in shaping microbial communities. For instance, studies have shown that soil amendments, such as biochar, can significantly enhance microbial diversity and community structure, as evidenced by increased Shannon indices in treated soils (Singh et al., 2022). This suggests that similar environmental interventions in the provinces could potentially influence the observed bacterial diversity. The differences in alpha diversity indices between the two provinces warrant further investigation. Factors such as soil composition, land use, and climatic conditions may play critical roles in shaping these microbial communities (Belus et al., 2022). For example, research indicates that higher plant diversity can enhance soil microbial diversity, suggesting a potential link between vegetation and microbial community structure (Li et al., 2023). This relationship may be particularly relevant in the context of KwaZulu-Natal and Limpopo, where varying land use practices and ecological conditions exist. In summary, the analysis of bacterial communities in KwaZulu-Natal and Limpopo reveals significant differences in microbial diversity, with Limpopo exhibiting higher diversity despite KZN having a greater abundance of bacterial communities. These findings underscore the importance of environmental factors in shaping microbial ecosystems and highlight the need for further research to explore the underlying mechanisms driving these differences.

#### 3.1.2 Beta diversity of the bacterial communities in the Limpopo and KwaZulu-Natal municipalities

The multi-dimensional scaling (MDS) using bray distance was employed to study the beta diversity of the bacterial communities in the KwaZulu-Natal and Limpopo provinces and municipalities. As illustrated in Figure 3A, the PC1 and PC2 accounted for 18.2% of the variance in the bacterial communities. The discrepancy in bacterial communities between the KwaZulu-Natal and Limpopo was also verified using a permutational ANOVA analysis (R^2^ = 0.11939, *p* < 0.001). This result is consistent with previous findings that the bacterial communities were different between KwaZulu-Natal and Limpopo provinces and municipalities. The KwaZulu-Natal and Limpopo samples were divided into two clusters (Figure 3B). Moreover, the Ward’s D2 distance hierarchical clustering tree branches of the samples collected from Mkhanyakude, KwaZulu-Natal had more cross-connections than the Limpopo samples. Although chicken faecal samples were sampled in different agroecological zones, the KwaZulu-Natal and Limpopo village chicken taxonomic clustered into separate clades, indicating that the bacterial similarity was closely related to the habitat type. The beta diversity network further illustrated that KwaZulu-Natal samples were closely cross-connected, and KwaZulu-Natal samples had a much closer network which signifies similarities in faecal microbiome composition. Overall, both the hierarchical clustering, MDS, and the beta diversity network analysis revealed visible differences in the bacterial communities between KwaZulu-Natal and Limpopo samples.

**Figure 3:**
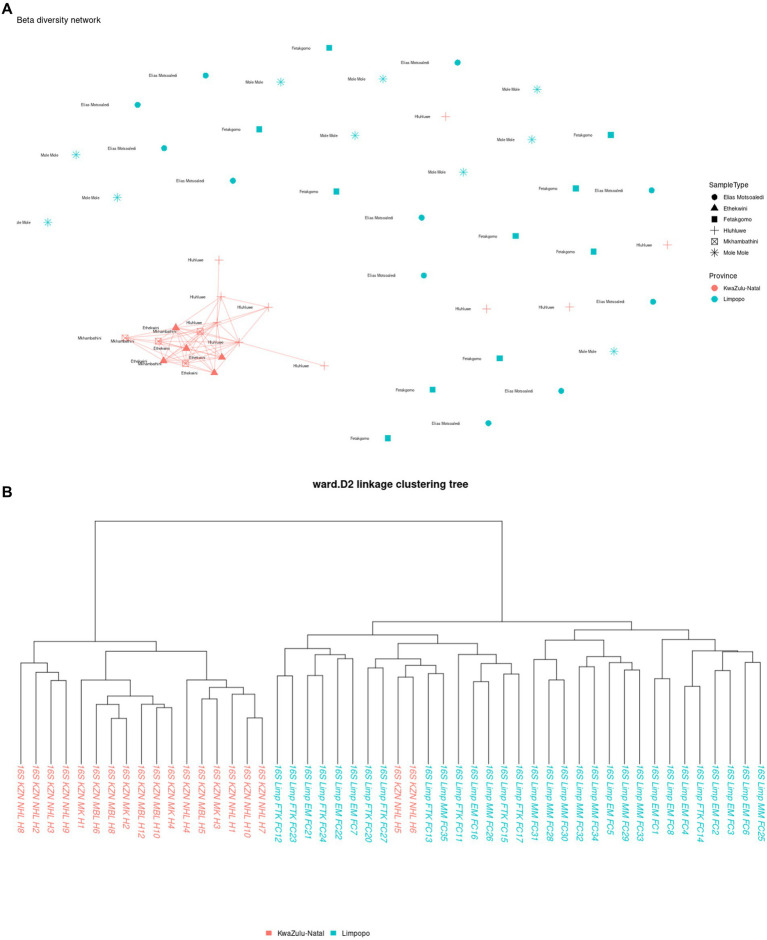
Beta diversity indices of KwaZulu-Natal and Limpopo village chicken faecal samples. **(A)** MDS diagram showing PC1 and PC2, which together accounted for 18.2% of the variance in the bacterial communities. PERMANOVA results indicate significant differences between provinces (R^2^= 0.11939, *P* < 0.001). Samples are grouped by province, with KwaZulu-Natal (red) and Limpopo (blue) forming distinct clusters, reflecting differences in microbial community composition. **(B)** Ward D2 distance linkage clustering dendrogram depicting the hierarchical relationships between samples based on 16S rRNA sequencing data. Samples from KwaZulu-Natal (red) and Limpopo (blue) are grouped, highlighting regional microbial community differences.

**Figure 3 (Continued):**
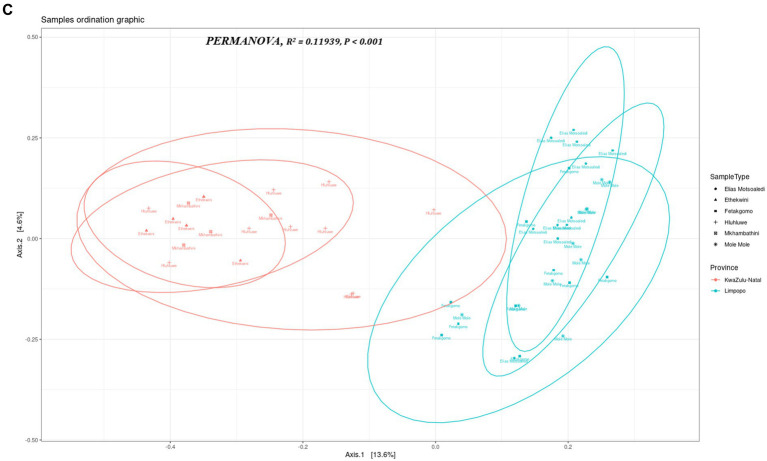
**(C)** Beta diversity network diagram based on Jaccard distance shows the clustering patterns of village chicken faecal samples. Samples from KwaZulu-Natal municipalities (red) exhibit a closely connected network, indicating high similarity in microbial community composition within the province. In contrast, Limpopo samples (blue) appear more dispersed, reflecting greater variation among municipalities. This highlights distinct beta diversity profiles between the two provinces.

The choice of analytical methods for assessing beta diversity in the faecal microbiome of village chickens from KwaZulu-Natal and Limpopo provinces was driven by the need to elucidate the differences in microbial community composition between these two regions. Beta diversity analysis is crucial for understanding the variation in microbial communities across different environments, and it provides insights into the ecological dynamics of these communities. The use of hierarchical clustering, multidimensional scaling (MDS), and beta diversity network analysis allows for a comprehensive examination of the microbial composition. Hierarchical clustering groups samples based on their similarities, enabling the identification of distinct clusters that reflect the underlying ecological relationships among the samples (Yu et al., 2021). This method is particularly effective in visualizing the similarities and differences in microbial communities, which can be influenced by factors such as geographical location and environmental conditions. Multidimensional scaling (MDS) complements hierarchical clustering by providing a visual representation of the data in reduced dimensions, making it easier to interpret complex relationships among samples (Hiltunen et al., 2021). This technique helps to highlight the proximity of samples within the same region, as seen in the KwaZulu-Natal samples, which exhibited a closer network, indicating similarities in their faecal microbiome composition. Such visualizations are essential for understanding how environmental factors may shape microbial communities in different regions.

In the context of microbial ecology, the comparison of bacterial communities across different geographical regions, such as KwaZulu-Natal and Limpopo in South Africa, provides invaluable insight into how environmental factors and habitat types shape community composition and diversity. The findings reported, show significant differences in beta diversity between the two provinces, underscore the complexities of microbial ecosystems and their responses to local conditions. The use of multi-dimensional scaling (MDS) along with Bray-Curtis distance is a robust approach for revealing community structure. The observed 18.2% variance explained by the first two principal components often necessitates a cautious interpretation, as it indicates that a considerable portion of the variance remains unexplained. Future research could focus on integrating additional environmental variables to capture more of the underlying ecological dynamics that drive community assembly. The significant results from the permutational ANOVA analysis (R^2^ = 0.11939, *p* < 0.001) further corroborate the distinctness of the bacterial communities in the two provinces. Such findings align with the broader body of literature that indicates geographical separation often leads to differentiation in microbial communities due to variations in climate, soil type, and anthropogenic influences (Peterson et al., 2022; Zhang et al., 2023). The clustering of the samples based on the agroecological zones further suggests that environmental contexts and habitat types play a crucial role in determining microbial composition. This is consistent with previous studies indicating that spatial factors and habitat specificities strongly influence microbial diversity (Liu et al., 2020). The hierarchical clustering results, which highlighted more cross-connections within KwaZulu-Natal samples compared to Limpopo, suggest that the bacterial communities in KwaZulu-Natal may have higher functional redundancy or shared ecological niches, potentially driven by higher resource availability or more stable environmental conditions (Nengovhela et al., 2021). Additionally, the separation of chicken faecal samples into distinct clades emphasizes the impact of habitat on microbial community structure. Previous research has demonstrated that host-related factors, such as dietary components and management practices, can significantly influence gut microbiota composition (Joat et al., 2023; Shang et al., 2021). Moreover, the beta diversity network analysis indicates a tighter network of connections among KwaZulu-Natal samples. This observation suggests that the bacterial communities in this region might have a more stable or resilient microbial ecosystem, which can provide insights into the implications for ecosystem health and host resilience. Understanding the relational dynamics within these communities can yield critical information, especially in light of the increasing stressors imposed by agricultural practices and climate change (Shade, 2023). The patterns observed in the bacterial community structures between KwaZulu-Natal and Limpopo provinces illuminate the intricate interplay between ecological factors and microbial diversity. Future investigations could benefit from a multi-faceted approach, combining metagenomic techniques and long-term ecological monitoring to further elucidate how specific environmental variables impact microbial community dynamics over time.

#### 3.1.3 Taxonomic composition of the bacterial communities in the Limpopo and KwaZulu-Natal municipalities

**Figure 4:**
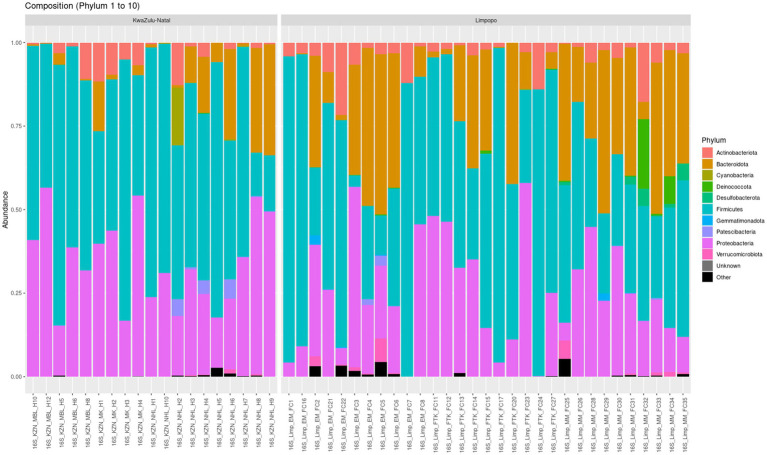
Taxonomic relative abundance of top 10 bacteria phyla from village chicken faecal microbiome from KwaZulu-Natal and Limpopo provinces.

A total of 8,982 taxa by 6 taxonomic ranks were detected in the KwaZulu-Natal and Limpopo village chicken faecal samples and were used for subsequent analysis. Microbial relative abundances at phylum and genus levels in the KwaZulu-Natal and Limpopo groups are shown in Figure 4, Supplementary Table 2, respectively. The phyla Proteobacteria, Firmicutes and Bacteroidetes, had higher relative abundance and were the dominant bacteria in both groups in the KwaZulu-Natal and Limpopo, accounting for over 99% of the microbial community, although the proportion of each genus differed between the KwaZulu-Natal and Limpopo samples. The genera *Escherichia, Shigella*, *Lactobacillus*, *Pseudomonas*, *Flavobacterium*, and *Pedobacter* had a higher relative abundance and were dominant in KwaZulu-Natal and Limpopo, respectively. Overall, both the relative abundances and the beta diversity network analysis revealed visible differences in the bacterial communities between KwaZulu-Natal and Limpopo samples which further emphasizes the hierarchical clustering, MDS and beta diversity network analysis findings that bacterial communities in between KwaZulu-Natal and Limpopo village chicken faecal samples vary significantly.

The identification of a diverse array of microbial taxa in the village chicken faecal samples from KwaZulu-Natal and Limpopo, amounting to 8,982 taxa across six taxonomic ranks, speaks to the complexity and richness of the gut microbiome in these agricultural settings. The dominance of the phyla Proteobacteria, Firmicutes, and Bacteroidetes, comprising over 99% of the microbial communities in both regions, is consistent with findings in previous studies highlighting these taxa as pivotal components of avian gut microbiomes (Sun et al., 2024). The relative abundances of the genera *Escherichia*, *Shigella*, *Lactobacillus*, *Pseudomonas*, *Flavobacterium*, and *Pedobacter* reveal important insights into potential ecological and health implications for poultry. The prevalence of *Escherichia*, particularly *E. coli*, is critical as it can be both beneficial and pathogenic, depending on the specific strains present. This duality raises concerns regarding biosecurity and the management of gut health in poultry production systems (Li et al., 2023). Furthermore, Lactobacillus is recognized for its probiotic properties, contributing to gut health and influencing gut microbiota composition (Dameshghian et al., 2024). The relative abundance of these genera may reflect various management practices, dietary influences, or environmental factors characteristic of poultry farming in the respective regions (Muyyarikkandy et al., 2023). The differences observed in the relative abundances at the genus level suggest that local environmental conditions, such as temperature, humidity, and availability of feed resources, likely impact the microbial communities (Cai et al., 2024).

The results from the beta diversity network analysis align with the hierarchical clustering and MDS findings, reinforcing the notion that the bacterial communities in KwaZulu-Natal and Limpopo are distinct. This separation is critical, as it suggests that the microbiomes may respond differently to health challenges, thus affecting production efficiency and disease resistance in village chickens (Wang et al., 2023; Tröscher-Mußotter et al., 2022). Understanding these differences can be integral to tailoring vaccination and management strategies aimed at enhancing poultry health and productivity.

Importantly, while this study sheds light on the microbial communities in poultry, it raises several questions for future research. Investigating the functional potential of these microbial communities via metagenomic sequencing could provide deeper insights into metabolic capabilities and interactions among microbes (Zhou et al., 2022). Additionally, longitudinal studies could help elucidate how these communities change in response to environmental shifts or management practices. The findings from this analysis underscores the importance of microbiome research in understanding the ecological dynamics of village chicken gut health. The evident disparities in bacterial community composition between KwaZulu-Natal and Limpopo highlight the influence of local conditions on microbial ecosystems, pointing to the need for contextualized approaches to poultry health management in South Africa.

### 3.2 Shotgun metagenomic data

#### 3.2.1 Sequencing and co-assembly statistics

Shotgun metagenomic sequencing of the 48 DNA samples from village chicken faecal samples in KwaZulu-Natal and Limpopo produced 594.9 million 150 bp paired-end reads, with an average of 49.6 million reads per sample and a range of 39.9 to 79.9 million reads between samples. After quality control, 529.9 million paired-end reads spanning in length from 50 to 140 bp and averaging 44.2 million per sample were retained, with a range of 34.8 to 72.9 million. The libraries in KwaZulu-Natal and Limpopo had 256.6 million and 273.3 million paired-end reads, respectively, after combining the reads by province.

The co-assembly of the Limpopo paired-end reads generated a total of 367,892 contigs (374,273 bp in the largest contig), with N50 of 3,480 bp and L50 of 106,965 bp, while for KwaZulu-Natal, it produced 366,749 contigs (515,562 bp in the largest contig), with N50 of 4,207 bp and L50 of 89,012 bp. A total of 19 KwaZulu-Natal and 39 Limpopo bins resulted from dereplication, aggregation and scoring strategy, DAS Tool (Sierber, et al., 2018) in which 11 and 30, respectively, passed our quality filter (≥50% of completeness and ≤ 10% of contamination).

The results from the shotgun metagenomic sequencing of village chicken faecal samples in KwaZulu-Natal and Limpopo reveal extensive insights into the microbial diversity and composition within these agricultural ecosystems. The production of 594.9 million 150 bp paired-end reads from 48 DNA samples underscores the high-throughput capability of metagenomic sequencing and its effectiveness in capturing a comprehensive snapshot of the microbial communities present in these settings. After quality control, retaining 529.9 million paired-end reads, with an average of 44.2 million reads per sample, indicates that the sequencing process was precise, yielding a robust dataset suitable for downstream analyses. The observed range in read counts (from 34.8 to 72.9 million per sample) reflects variations in microbial load or diversity among individual faecal samples, which may be influenced by factors such as diet, environment, and management practices (Shang et al., 2021; Chen et al., 2023).

The co-assembly of paired-end reads yielded a significant number of contigs for both provinces, with Limpopo producing 367,892 contigs and KwaZulu-Natal generating 366,749 contigs. The largest contigs from both datasets (374,273 bp for Limpopo and 515,562 bp for KwaZulu-Natal) provide valuable sequences that can be annotated for functional potential and taxonomic classification. The N50 and L50 values are critical metrics for evaluating the quality of assembly; for instance, the N50 of 4,207 bp in KwaZulu-Natal indicates a higher average contig length compared to Limpopo’s 3,480 bp, suggesting that KwaZulu-Natal samples may harbor more complex or diverse microbial genomes (Xiong et al., 2023). The generation of forest and pasture bins from the DAS Tool indicates a systematic approach to binning contiguous sequences into taxonomic units. The fact that 11 KwaZulu-Natal bins and 30 Limpopo bins passed the quality filter (≥50% completeness and ≤10% contamination) is promising; these bins represent distinct ecological niches and can be pivotal in understanding the functional dynamics of the microbial communities in different habitat types (de Vries et al., 2023; Tröscher-Mußotter et al., 2022).

Furthermore, the results reflect how ecological factors, such as land use and habitat type, influence microbial community structure and function (Jovel et al., 2016). With the observed discrepancies in contig lengths and bin compositions between the two provinces, implications for chicken health, nutrition, and overall farm management are significant. These findings align with recent research indicating that variations in bacterial communities can directly affect poultry health and productivity, highlighting the importance of habitat and environmental management in sustainable agricultural practices (Zhang et al., 2023). The metagenomic data produced from village chicken faecal samples in KwaZulu-Natal and Limpopo illustrate the microbial richness and diversity in these regions. The differences in contig characteristics and the successful assembly of quality bins point towards a complex interplay of ecological factors that could inform future research on poultry gut health and microbial ecology strategies to enhance the productivity and sustainability of chicken farming in South Africa.

#### 3.2.2 Taxonomic assignment and relative abundance of metagenome-assembled genomes

The taxonomic classification of the Limpopo province village chicken faecal microbiome resulted in 61 bins belonging to 15 bacterial phyla: Actinobacteria, Proteobacteria, Firmicutes, Firmicutes A, Bacteriodota, Desulfobacterota, Desulfobacterota, Elusimicrobia, Spirochaetota, Verrucomicrobiota, Deinococcota, Myxococcota, Gemmatimonadota, Planctomycetota, and Synergistota (Supplementary Table 3). Twenty-six bins could be classified at the species level. KwaZulu-Natal province village chicken faecal microbiome bins, 30 belonged to 7 bacterial phyla: Actinobacteria, Proteobacteria, Firmicutes, Bacteriodota, Campylobacterota, Firmicutes A and Firmicutes D (Supplementary Table 4). Only eight bins could be classified at the species level, thus demonstrating the potential of this approach to reveal the yet-unknown microbial diversity of the KwaZulu-Natal village chicken faecal microbiome. The prevalent phyla based on iTOL revealed a high prevalence of Proteobacteria, Actinobacteria, Firmicutes, Verrucomicrobiota, Bacteriodota, Synergistota, Spirochaetota, and Gemmatimonadota respectively, for the Limpopo province village chicken faecal microbiota (Figure 5A). The Kwazulu—Natal village chicken faecal microbiota prevalent phyla based on iTOL, included Proteobacteria, Bacteriodota, Planctomycetota, Verrucomicrobiota, and Actinobacteria, respectively. The microbiota of village chickens from KwaZulu-Natal also exhibited a prevalence of candidate phyla that have not yet been fully cultured or sequenced (Figure 5B, Dunfield et al., 2012), including Candidatus division Zixibacter, Candidatus Cloacimonadota, Candidatus Aureabacteria, Candidatus Fermentibacteria, Candidatus Aegribacteria, Candidatus Saganbacteria, Candidatus Sericytochromatia, Candidatus Gastanaerophilales, and Candidatus division WS1. Overall, the taxonomic assignment of the village chicken faecal microbiome from Limpopo and KwaZulu-Natal reveals some similarities in prevalent phyla, however, KwaZulu—Natal province depicts a greater variation and diversity in the microbiota including numerous candidate phyla, which has not been fully characterised as yet.

**Figure 5:**
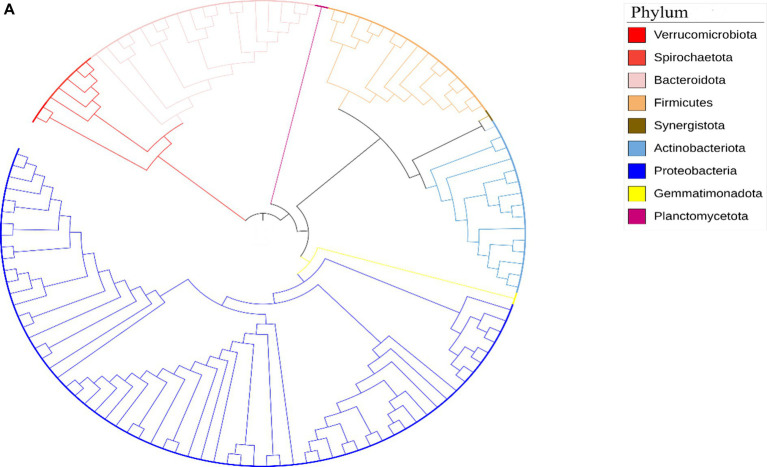
**(A)** Circular phylogenetic tree representing the taxonomic diversity of bacterial phyla identified in chicken fecal microbiota samples from the Limpopo region. The tree was constructed using Mega software and visualized with iTOL (Interactive Tree of Life). The different phyla are color-coded according to the legend: Verrucomicrobiota (red), Spirochaetota (salmon), Bacteroidota (light pink), Firmicutes (orange), Synergistota (brown), Actinobacteriota (light blue), Proteobacteria (dark blue), Gemmatimonadota (yellow), and Planctomycetota (purple). The branching pattern demonstrates the evolutionary relationships between the different bacterial groups, with the length of branches indicating the degree of genetic divergence. This analysis reveals the complex microbial community structure present in the chicken gut microbiome from this geographical region.

**Figure 5 (Continued):**
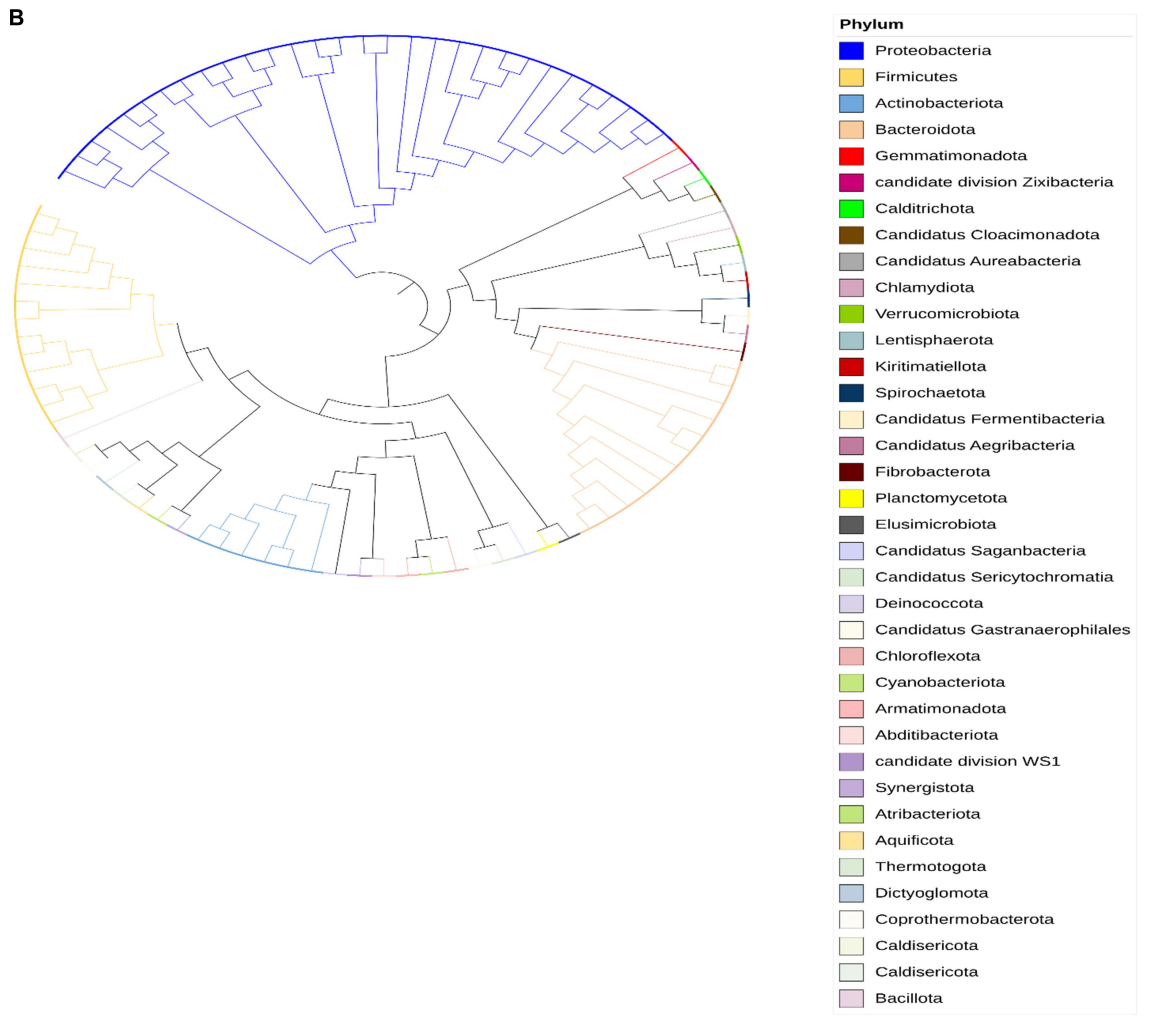
**(B)** Circular phylogenetic tree representing the taxonomic composition of bacterial communities in chicken fecal microbiota from KwaZulu Natal, analyzed using shotgun metagenomic sequencing data. The tree was constructed and visualized using Mega and iTOL (Interactive Tree of Life) software. The colors on the nodes indicate different bacterial phyla as shown in the legend, including dominant groups such as Proteobacteria (blue), Firmicutes (yellow), Actinobacteriota (light blue), and Bacteroidota (peach), along with numerous candidate phyla and less abundant groups. The branching patterns illustrate the evolutionary relationships between the identified bacterial taxa, with branch lengths representing genetic distances. This analysis reveals a complex and diverse microbial community structure, with multiple bacterial phyla and candidate divisions present in the chicken gut microbiome from this geographical region. The presence of multiple candidate phyla and newly described bacterial groups highlights the potential for novel microbial diversity in these samples.

The taxonomic analysis of the village chicken faecal microbiome from Limpopo and KwaZulu-Natal provinces reveals interesting contrasts and insights into the microbial diversity influenced by geographical and environmental factors. The detection of 15 distinct bacterial phyla in Limpopo compared to 7 in KwaZulu-Natal underscores the regional differences in microbial diversity, with Limpopo exhibiting a broader range of bacterial taxa. However, despite the smaller number of phyla identified in KwaZulu-Natal, the presence of numerous candidate phyla highlights a potentially unique and understudied microbiota in this region.

In Limpopo, prevalent phyla such as Proteobacteria, Firmicutes, Actinobacteria, Verrucomicrobiota, Bacteroidota, Synergistota, Spirochaetota, and Gemmatimonadota suggest a microbiome reflective of the agricultural and environmental conditions of the province. The dominance of Proteobacteria and Firmicutes is consistent with findings from other studies examining poultry microbiomes, where these phyla play essential roles in nutrient digestion and pathogen defense (Waite and Taylor, 2014). Proteobacteria are often associated with diverse metabolic functions, which may provide chickens in Limpopo with the ability to adapt to environmental changes and resource variability (Lozupone et al., 2012).

The microbiome of KwaZulu-Natal chickens, while represented by fewer phyla, exhibits a high proportion of candidate phyla, including Candidatus divisions such as Zixibacter, Cloacimonadota, and Aureabacteria. These candidate phyla, which remain poorly characterized, could represent unique ecological niches within KwaZulu-Natal’s local environment. The presence of these uncultivated and potentially novel microbial taxa suggests that the KwaZulu-Natal microbiome could be shaped by unique ecological pressures, potentially due to climatic differences or distinct management practices compared to Limpopo (Dunfield et al., 2012). This finding is particularly relevant as candidate phyla often include microorganisms that exhibit specialized metabolic capabilities, such as anaerobic processes, which can play crucial roles in complex microbial ecosystems (Hug et al., 2016). The discovery of these candidate phyla also aligns with the broader body of literature on microbial biogeography, where environmental and spatial variables can drive the divergence of microbial community composition (Martiny et al., 2006).

The fact that only 26 bins in Limpopo and 8 in KwaZulu-Natal could be classified at the species level demonstrates both the limitations and potential of taxonomic binning in revealing microbial diversity in unexplored ecosystems. These findings underscore the under-representation of many bacterial taxa in existing databases, especially for environmental samples like village chicken fecal microbiota. Expanding microbial reference databases could improve species-level identification and uncover additional microbial functions relevant to host health and adaptation.

Overall, the prevalence of shared phyla such as Proteobacteria, Actinobacteria, and Bacteroidota between the provinces suggests a core microbial community potentially essential for the host’s metabolic and immune functions (Garinet et al., 2018; Yao et al., 2018). However, the unique microbiome diversity observed in KwaZulu-Natal, particularly the abundance of candidate phyla, points to a more varied microbial ecosystem that warrants further investigation. Expanding research to focus on the functional roles of these candidate phyla could reveal novel insights into how local conditions influence microbial community assembly, resilience, and host health.

#### 3.2.3 Functional characterization of MAGs and biogeochemical relevance

The gut microbiome plays a crucial role in the health and adaptability of extensively raised chickens, particularly in low-resource production systems like those found in Limpopo and KwaZulu-Natal, South Africa (Figures 6A,B). This study reveals significant insights into the metabolic pathways present in the intestinal microbiota, highlighting how these microorganisms adapt to their environments. We identified genes related to critical metabolic pathways, including glycolysis (Embden-Meyerhof pathway), the pentose phosphate pathway, citrate (TCA or Krebs cycle), glyoxylate, reductive pentose phosphate (Calvin cycle), reductive citrate (Arnon-Buchanan cycle), and dicarboxylate-hydroxybutyrate cycles across all metagenome-assembled genomes (MAGs), with notable variability between the two regions. This variability suggests ecological adaptations specific to each region. Our findings align with recent research indicating that many prokaryotes utilize alternative pathways like the Entner-Doudoroff (ED) pathway instead of the canonical EMP pathway. Anaerobes typically favour the higher ATP yield of the EMP pathway, while aerobes, such as those identified in our study, more often employ the ED pathway due to its lower protein cost (Flamholz et al., 2013). The presence of the full pathways for glycolysis, the pentose phosphate pathway, and ED pathways indicates that gut microorganisms can flexibly adapt their metabolism based on oxygen levels and nutritional content. Interestingly, we found no complete carbon fixation pathways within the MAGs, indicating reliance on dietary inputs rather than autotrophic carbon fixation, which could provide insights into the nutritional strategies of village chickens in low-resource settings (Sieber et al., 2018). Furthermore, the identification of several electron transport chain complexes (I-V) associated with aerobic respiration suggests that the gut environment is conducive to aerobic microbial communities, potentially impacting energy metabolism and overall gut health.

**Figure 6:**
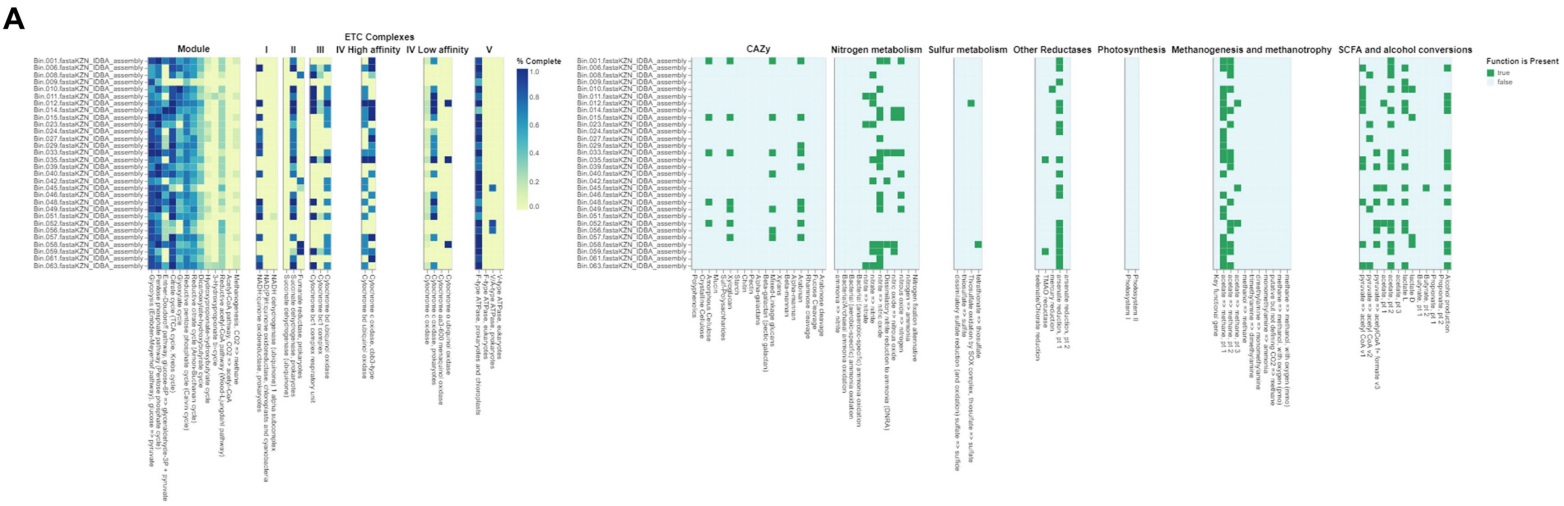
**(A)** DRAM (Distilled and Refined Annotation of Metabolism) functional annotations of bacterial genomes from Limpopo chicken fecal microbiota. The heatmap displays the distribution and completeness of various metabolic pathways and functional modules across different bacterial assemblies (Bin_001 through Bin_063). The left panel shows ETC (Electron Transport Chain) complexes I-V with color intensity indicating completeness (0.0-1.0, yellow to blue). The right panels illustrate the presence (green) or absence (white) of key metabolic functions including CAZy (Carbohydrate-Active Enzymes), nitrogen metabolism, sulfur metabolism, other reductases, photosynthesis, methanogenesis and methanotrophy, and SCFA (Short-Chain Fatty Acid) and alcohol conversions. This comprehensive metabolic profiling reveals the functional potential of the microbial communities in the chicken gut ecosystem from the Limpopo region.

**Figure 6 (Continued):**
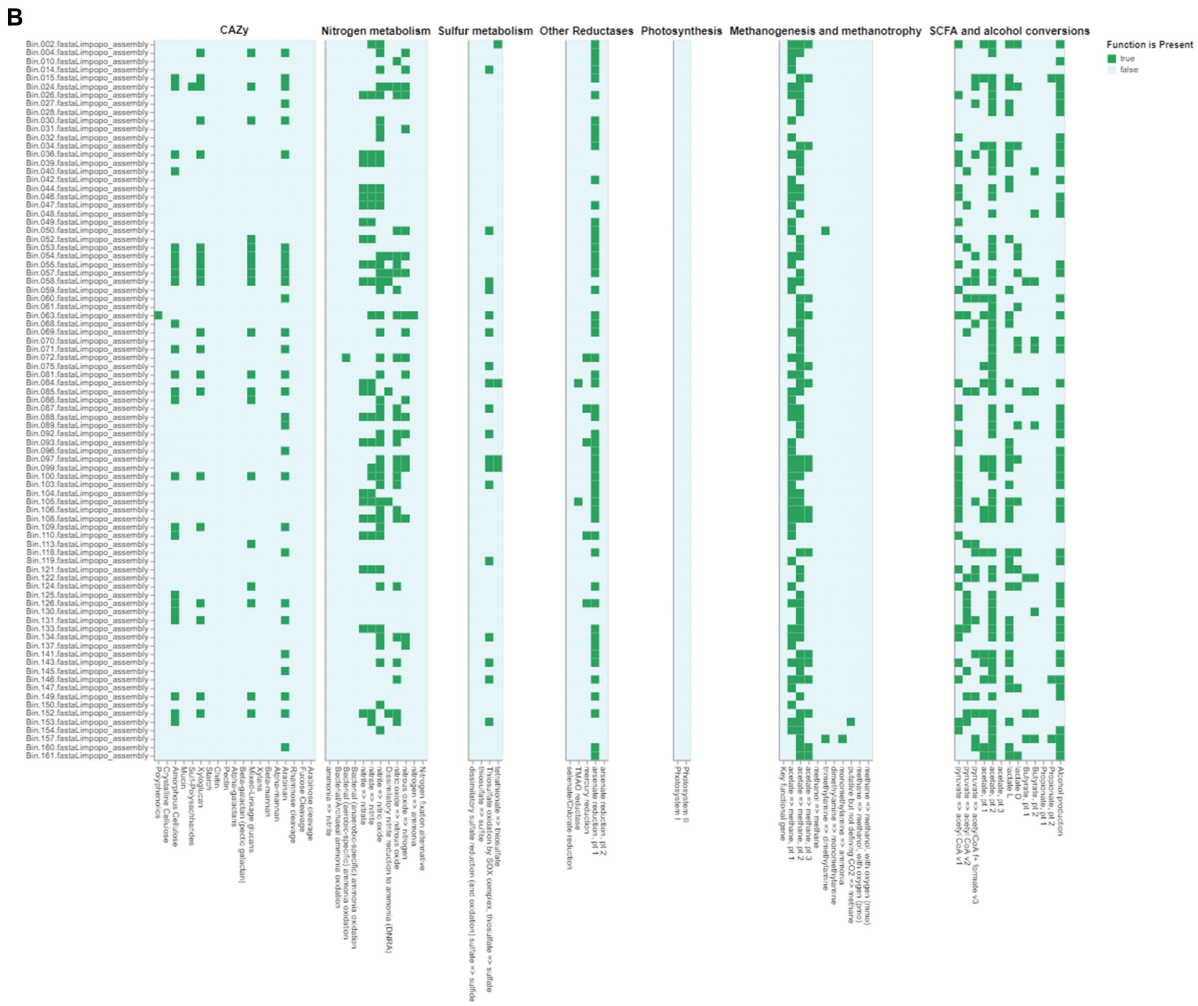
**(B)** Functional annotation of metagenome-assembled genomes (MAGs) from KwaZulu Natal chicken fecal samples using DRAM (Distilled and Refined Annotation of Metabolism). The MAGs were selected based on their completeness (≥90%) and contamination (≤5%). The presence (green) or absence (white) of key metabolic functions is shown across different bacterial assemblies (Bin_002 through Bin_161). The heatmap displays the distribution of various functional categories including CAZy (Carbohydrate-Active Enzymes), nitrogen metabolism, sulfur metabolism, other reductases, photosynthesis, methanogenesis and methanotrophy, and SCFA (Short-Chain Fatty Acid) and alcohol conversions. Each row represents an individual MAG, while columns represent specific metabolic functions and pathways. This analysis provides insights into the metabolic capabilities and functional potential of the bacterial communities present in the chicken gut microbiome from the KwaZulu Natal region, highlighting the diversity of metabolic pathways present across different bacterial taxa.

Recent studies have shown that various metabolic pathways exist in microorganisms, reflecting diverse adaptations to environmental conditions. For example, research by Sato and Atomi (2011) on Archaea identified modified glycolytic pathways and novel CO2-fixing pathways, which may parallel similar adaptations in the gut microbiomes of village chickens. Additionally, comparative analyses of lactic acid bacteria have revealed lineage-specific trends in gene loss and gain within glycolytic and pentose phosphate pathways (Salvetti et al., 2013). Such findings may shed light on the metabolic versatility of the gut microbiota identified in this study. Research on Actinobacteria has also demonstrated variability in upper glycolytic pathways with conservation in lower pathways, suggesting potential pathways for metabolic adaptation (Verma et al., 2013). The presence of Actinobacteria in our MAGs indicates their potential significance in carbohydrate metabolism and health outcomes for village chickens. Overall, this study underscores the critical role of the gut microbiome in the health and adaptability of village chickens, linking microbial diversity to production efficiency in low-resource settings. Future research should investigate archaeal populations’ contributions to gut metabolism and assess the impact of specific metabolic pathways on health outcomes to better understand microbial ecology in agricultural systems.

#### 3.2.4 Antibiotic resistome profiles from KwaZulu-Natal and Limpopo provinces

##### 3.2.4.1 Antibiotic resistome from Limpopo Province

The sensitivity of detection for Limpopo, which is the ability of the genotypic test to detect antimicrobial resistance (true positive rate), was >90% for 3 antimicrobials: streptomycin, tetracycline, erythromycin, and azithromycin. The following ARO genes were detected, *ant(6)-Ia, tet(36)*, and *msr(C)*. Based on the CARD database (Appendix 6), the sensitivity of detection, which is the ability of the genotypic test to detect antimicrobial resistance (true positive rate), was assessed for 24 bins from Limpopo using RGI criteria (perfect and strict). The detected resistance included *aminoglycosides*, *aminocoumarins*, *phosphonic acids*, *macrolides*, *fluoroquinolones*, *carbapenems*, *cephalosporins*, *glycylcyclines*, *cephamycins*, *penams*, *tetracyclines*, *peptides*, *rifamycins*, *phenicols*, *penems*, *nucleosides*, and *nitroimidazoles*, as well as disinfecting agents and antiseptics. These results indicate a diverse range of antimicrobial resistance profiles among the samples from Limpopo. The following ARO genes were detected, *cpxA, mdtG, TolC, emrR, msbA, marA, baeS, baeR, AcrE, evgA, emrY, Escherichia coli acrA, H-NS, mdtP, EC-13, mdtH, mdtA, emrA. emrB, Escherichia coli emrE, kdpE, rsmA, AcrS, leuO,* and *mdtN*. The STARAMR and CARD database detection results for Limpopo province, both revealed the following resistomes with perfect matches, *Enterococcus faecalis, Staphylococcus aureus*, *Listeria innocua*, *Clostridium botulinum*, *Herbinix luporun*, *Staphylococcus aureus,* and *Enterococcus faecium.* However, the CARD database revealed the *Escherichia coli* resistome, based on the fluoroquinolone antibiotic detection using RGI, which also revealed high prevalence of *E. coli* in Limpopo.

The antimicrobial resistance (AMR) profile of the Limpopo village chicken faecal microbiome demonstrates a high sensitivity of detection for resistance to several commonly used antimicrobials, notably streptomycin, tetracycline, erythromycin, and azithromycin. The detection rate, which exceeded 90%, reflects the efficacy of genotypic testing in identifying resistance to these antimicrobials. The specific resistance genes detected—such as *ant(6)-Ia*, *tet(36)*, and *msr(C)*—highlight the presence of aminoglycoside, tetracycline, and macrolide resistance, respectively. These findings are significant, as they indicate the potential for resistance transmission to pathogens of both veterinary and public health concern.

The diverse resistance gene profile detected through the CARD database and RGI criteria (with “perfect” and “strict” matches) in 24 bins from Limpopo further underscores the complexity of resistance patterns in this region. The presence of resistance mechanisms against a wide array of antibiotic classes, including aminoglycosides, macrolides, carbapenems, and cephalosporins, among others, highlights the adaptability of the microbiome to various selective pressures, possibly resulting from environmental exposure to antimicrobial agents used in agriculture (Kraemer et al., 2019). Notably, genes associated with resistance to disinfectants and antiseptics (e.g., *msbA*, *TolC*, *mdtG*) suggest that microbial populations in Limpopo may have been exposed to biocides commonly used in livestock management or environmental sanitation, potentially contributing to the observed resistance diversity (Buffet-Bataillon et al., 2012). Interestingly, the presence of genes such as *marA* and *acrA*, which are associated with multi-drug efflux pumps, suggests a mechanism by which bacteria in the Limpopo microbiome could resist multiple antimicrobial agents, effectively enhancing their survival in environments with diverse antibiotic exposures (Blair et al., 2015). The CARD and STARAMR detection results reveal a range of resistomes among species typically associated with both animal and human microbiomes, including *Enterococcus faecalis*, *Staphylococcus aureus*, and *Escherichia coli*. The detection of *E. coli* resistance to fluoroquinolones is particularly notable, as it indicates a high prevalence of this organism in Limpopo samples and underscores potential risks to public health given fluoroquinolones’ critical role in treating severe bacterial infections in humans (Patel et al., 2020).

The overlap between CARD and STARAMR database detections with “perfect” matches for resistant strains (e.g., *Staphylococcus aureus*, *Enterococcus faecium*) highlights the reliability of these databases for identifying clinically relevant AMR genes and further reinforces the value of molecular AMR profiling in characterizing environmental reservoirs of resistance. The detection of *Clostridium botulinum* and *Listeria innocua* resistomes, while less common, adds another layer of potential concern given these organisms’ known pathogenicity and their association with foodborne illnesses. The AMR gene diversity observed in Limpopo village chicken microbiota reflects a potentially significant reservoir of resistance, with implications for both poultry health and zoonotic transmission to humans (Cho and Blaser, 2021). This diverse resistome not only highlights the need for monitoring AMR in agricultural settings but also reinforces the value of genotypic surveillance using databases like CARD and STARAMR to assess resistance risks and inform responsible antimicrobial stewardship practices in the region (Table 1).

**Table 1:** Antibiotic resistome profile from Limpopo province village chicken faecal microbiome.

ARO Term	Predicted phenotype	Resistomes with perfect matches	Resistance mechanism	%Identity
ant(6)-Ia	Streptomycin	*Enterococcus faecalis, Staphylococcus aureus, Listeria innocua*	Antibiotic inactivation	100
tet(36)	Tetracycline	*Clostridium botulinum, Herbinix luporun, Staphylococcus aureus*	Antibiotic target protection	98.80
msr(C)	Erythromycin, azithromycin	*Enterococcus faecium*	Antibiotic target protection	98.85

#### 3.2.5 Antibiotic resistome profile from KwaZulu-Natal province

The sensitivity of detection for KwaZulu-Natal, which is the ability of the genotypic test to detect antimicrobial resistance (true positive rate), was >90% for 4 antimicrobials: gentamicin, tetracycline, erythromycin, azithromycin and lincomycin. The following ARO genes were detected: *aac(6′)-Iid*, *mph(A)*, *tet(Z)*, and *lsa(A)*. Based on the CARD database (Appendix 7), the sensitivity of detection—indicating the ability of the genotypic test to detect antimicrobial resistance (true positive rate)—was over 90% for four bins from KwaZulu-Natal using RGI criteria (perfect and strict), covering resistance to *fluoroquinolones*, *monobactams*, *carbapenems*, *cephalosporins*, *glycylcyclines*, *cephamycins*, *penams*, *tetracyclines*, *peptides*, *aminoglycosides*, *rifamycins*, *phenicols*, *penems*, and disinfecting agents and antiseptics. The following ARO genes were detected, *marA, emrR, PmrF, AcrE, AcrS, YojI,* and *acrD.* The STARAMR and CARD database detection results for KwaZulu-Natal province, both revealed the following resistomes with perfect matches, *Enterococcus hirae, Salmonella enterica, Shigella flexneri, Vibrio cholerae, Escherichia coli, Corynebacterium glutamicum*, and *Enterococcus faecalis*.

The antimicrobial resistance (AMR) profile of the KwaZulu-Natal village chicken faecal microbiome demonstrates a high sensitivity of detection for resistance to five key antimicrobials: gentamicin, tetracycline, erythromycin, azithromycin, and lincomycin. With detection rates exceeding 90%, this indicates a robust capacity of the genotypic testing method to identify resistance genes, such as *aac(6′)-Iid*, *mph(A)*, *tet(Z)*, and *lsa(A)*, which are associated with resistance to aminoglycosides, macrolides, tetracyclines, and lincosamides, respectively. These findings are significant in understanding the distribution of specific AMR determinants within the KwaZulu-Natal microbiome and the implications for antimicrobial use in agriculture. Further insights from the CARD database and RGI criteria analysis underscore the diversity of resistance profiles within the KwaZulu-Natal microbiome. The testing revealed over 90% detection accuracy across four distinct microbiome bins, covering resistance to a broad range of antimicrobial classes, including fluoroquinolones, carbapenems, cephalosporins, and resistance to disinfectants and antiseptics. This wide range of resistance suggests exposure to various selective pressures, potentially from agricultural antimicrobials and biocides commonly used in animal farming (Martinez, 2009).

The identification of resistance genes linked to efflux pumps, such as *marA*, *acrD*, and *emrR*, suggests a potential mechanism that enables multi-drug resistance (MDR) by expelling diverse antimicrobial agents from bacterial cells, thereby supporting survival in environments exposed to antibiotics (Fernandes et al., 2015). Additionally, the presence of genes such as *PmrF* and *YojI*, which confer resistance to antimicrobial peptides and antibiotics, highlights adaptive responses that could pose challenges in treating infections caused by these bacteria (Nikaido, 2009). Results from both the STARAMR and CARD databases showed “perfect” matches in the resistomes of several clinically relevant bacteria in KwaZulu-Natal, including *Enterococcus hirae*, *Salmonella enterica*, *Shigella flexneri*, *Vibrio cholerae*, *Escherichia coli*, *Corynebacterium glutamicum*, and *Enterococcus faecalis*. These findings are critical, given the potential health risks associated with the transfer of AMR genes to pathogenic bacteria in human populations (Table 2).

**Table 2:** Antibiotic resistome profile from KwaZulu—Natal province village chicken faecal microbiome.

ARO Term	Predicted phenotype	Resistomes with perfect matches	Resistance mechanism	%Identity
aac(6′)-Iid	Gentamicin	*Enterococcus hirae*	Antibiotic inactivation	100
mph(A)	Erythromycin, azithromycin	*Salmonella enterica, Shigella flexneri, vibrio cholera, Escherichia Coli*	Antibiotic inactivation	99.67
tet(Z)	Tetracycline	*Corynebacterium glutamicum*	Antibiotic efflux	100
lsa(A)	Lincomycin	*Enterococcus faecalis*	Antibiotic target protection	98.33

## 4 Conclusion

This study reveals significant variations in the faecal microbiome of village chickens between KwaZulu-Natal and Limpopo provinces, with critical implications for public health. Analysis of bacterial composition indicates a high prevalence of pathogenic genera, including *Escherichia* and *Shigella*, raising concerns about potential zoonotic diseases outbreaks. The 16S rRNA gene sequencing data demonstrates that agroecological zones and scavenging production systems notably influence faecal microbiome composition. On the other hand, shotgun metagenomic sequencing uncovered distinct taxonomic compositions and metabolic functions, highlighting unique metabolic pathway profiles for metagenome-assembled genomes (MAGs) across the provinces. The rich microbiome diversity reflects the birds’ adaptation to their natural environment, essentially creating more resilient but possibly less productive birds. The antibiotic resistome profiles illustrate a diverse range of resistance genes, including key groups such as *Enterococcus*, *Salmonella*, *Shigella*, and *Staphylococcus*, with KwaZulu-Natal chickens exhibiting resistomes associated with *Enterococcus hirae*, *Salmonella enterica*, *Shigella flexneri*, *Vibrio cholerae*, and *Escherichia coli*. In contrast, Limpopo chickens show a higher prevalence of resistomes, including *Enterococcus faecalis*, *Staphylococcus aureus*, *Listeria innocua*, and *Clostridium botulinum*. The greater abundance and diversity of resistomes in Limpopo suggest an increased antimicrobial usage, emphasising the urgent need for training farmers on use and misuse of antimicrobials as well as monitoring antimicrobial resistance in these populations. The analysis of bacterial composition in the faeces of village chickens has shown a high prevalence of pathogenic genera such as *Escherichia* and *Salmonella*, which are known to be associated with zoonotic diseases Zishiri et al. (2016). The presence of these pathogens raises concerns about the potential for outbreaks of diseases that can be transmitted from chickens to humans, particularly in communities where close contact with poultry is common. Furthermore, the study’s use of 16S rRNA gene sequencing has demonstrated that the gut microbiome’s composition varies significantly between the two provinces, suggesting that local environmental conditions and farming practices play a crucial role in shaping these microbial communities (Rausch et al., 2019; Malatji et al., 2016).

In addition to pathogenic bacteria, the study highlights the presence of a diverse array of antibiotic resistance genes within the gut microbiota of village chickens. The resistome profiles indicate that chickens from KwaZulu-Natal exhibit resistance genes associated with *Enterococcus hirae*, *Salmonella enterica*, and *Escherichia coli*, while those from Limpopo show a higher prevalence of *Enterococcus faecalis* and *Staphylococcus aureus* (Akinola et al., 2019). The greater abundance and diversity of these resistance genes in Limpopo suggest a higher level of antimicrobial usage, which underscores the urgent need for educational initiatives aimed at farmers regarding the responsible use of antimicrobials and the monitoring of antimicrobial resistance (Walsh, 2018; Khanyile et al., 2015). This study underscores the necessity for further investigation into the functional aspects of the faecal microbiome and its interactions with environmental factors across different seasons, which could enhance our understanding of the ecological and health implications as well as temporal dynamics of these microbial communities. Future studies should also address limitations such as sample size and geographical representation to provide a more balanced view of the findings.

## Data Availability

The sequencing data supporting this study have been deposited in the NCBI Sequence Read Archive (SRA) under the BioProject ID PRJNA1180228. The data are publicly accessible and include all raw sequence files used in the analysis.
