# Supplementary material for: Profiling the diversity of the village chicken faecal microbiota using 16S rRNA gene and metagenomic sequencing data to reveal patterns of gut microbiome signatures
Source: Front Microbiol. 2025 Feb 4;15:1487595. doi: 10.3389/fmicb.2024.1487595 (PMC11832711; doi:10.3389/fmicb.2024.1487595)
Supplement: Supplementary file 1 [file Data_Sheet_1.docx]

Supplementary Material

Profiling the diversity of the village chicken faecal microbiota using 16S rRNA and metagenomic shotgun sequencing data to reveal patterns of gut microbiome signatures

**Mxolisi Nene^1, 2*^, Nokuthula Winfred Kunene^1^, Rian Pierneef^3,4,5^, Khanyisile Hadebe^2^**

^1^Department of Agriculture, University of Zululand, Private Bag X1001, KwaDlangezwa, 3886

^2^Biotechnology Platform, Agricultural Research Council, Private Bag X5, Ondersterpoort, 0110, South Africa

^3^Department of Biochemistry, Genetics and Microbiology, University of Pretoria, Pretoria, 0001, South Africa

^4^Centre for Bioinformatics and Computational Biology, University of Pretoria, Pretoria, 0001, South Africa

^5^DSI/NRF SARChI in Marine Microbiomics, Department of Biochemistry, Genetics and Microbiology, University of Pretoria, Pretoria, 0001, South Africa

# * Correspondence: Mxolisi Nene [NeneM@arc.agric.za](mailto:NeneM@arc.agric.za)

# Supplementary Data

# Supplementary Figures and Tables

## Supplementary Figures


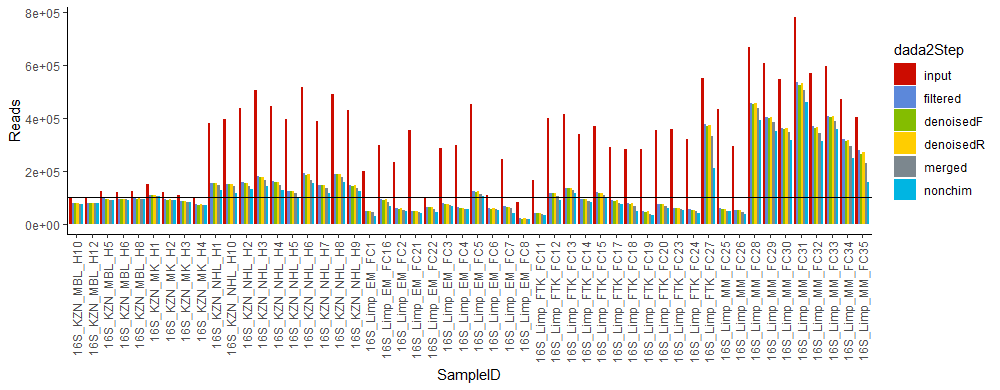


**Supplementary Figure 1:** Dada2 steps diagram, showing input, filtered, denoised forward, denoised reverse, merged, and no chimeric reads for KwaZulu-Natal and Limpopo village chicken fecal microbiome.

**1.2 Supplementary Tables**

| **SAMPLE** | **Observed** | **Chao1** | **se.chao1** | **ACE** | **se.ACE** | **Shannon** | **Simpson** | **InvSimpson** | **Fisher** |
| --- | --- | --- | --- | --- | --- | --- | --- | --- | --- |
| X16S_KZN_MBL_H10 | 217 | 217 | 0 | 217 | 6,2 | 3,41 | 0,9 | 10,43 | 27,45 |
| X16S_KZN_MBL_H12 | 218 | 218 | 0,12 | 218,22 | 4,84 | 3,07 | 0,79 | 4,78 | 27,5 |
| X16S_KZN_MBL_H5 | 513 | 513,11 | 0,41 | 513,34 | 9,93 | 4,25 | 0,96 | 22,49 | 71,95 |
| X16S_KZN_MBL_H6 | 184 | 184 | 0,25 | 184,15 | 4,85 | 3,24 | 0,92 | 11,89 | 22,15 |
| X16S_KZN_MBL_H8 | 184 | 184 | 0 | 184 | 5,14 | 3,3 | 0,91 | 10,57 | 22,1 |
| X16S_KZN_MK_H1 | 459 | 459,86 | 1,4 | 460,02 | 6,79 | 4,69 | 0,97 | 33,07 | 61,86 |
| X16S_KZN_MK_H2 | 301 | 301 | 0 | 301 | 6,35 | 3,54 | 0,89 | 8,94 | 38,99 |
| X16S_KZN_MK_H3 | 183 | 183,6 | 1,19 | 184,16 | 4,53 | 3,63 | 0,94 | 17,3 | 22,33 |
| X16S_KZN_MK_H4 | 350 | 350 | 0,17 | 350,15 | 7,17 | 3,68 | 0,89 | 9,07 | 48,07 |
| X16S_KZN_NHL_H1 | 152 | 152 | 0 | 152 | 5,03 | 2,44 | 0,8 | 5,01 | 17,07 |
| X16S_KZN_NHL_H10 | 129 | 129 | 0 | 129 | 5,05 | 2,6 | 0,88 | 8,14 | 14,33 |
| X16S_KZN_NHL_H2 | 738 | 738 | 0 | 738 | 12,84 | 3,99 | 0,95 | 19,49 | 103,17 |
| X16S_KZN_NHL_H3 | 450 | 450 | 0,02 | 450,26 | 8,99 | 3,74 | 0,94 | 16,7 | 57,52 |
| X16S_KZN_NHL_H4 | 624 | 624,33 | 0,7 | 624,92 | 10,08 | 4,55 | 0,96 | 28,46 | 85,38 |
| X16S_KZN_NHL_H5 | 168 | 168 | 0 | 168 | 5,03 | 3,88 | 0,97 | 29,19 | 19,77 |
| X16S_KZN_NHL_H6 | 724 | 724,12 | 0,38 | 724,73 | 10,45 | 4,99 | 0,98 | 60,88 | 98,57 |
| X16S_KZN_NHL_H7 | 253 | 253 | 0 | 253 | 6,91 | 3,12 | 0,91 | 11,06 | 30,72 |
| X16S_KZN_NHL_H8 | 516 | 516,06 | 0,26 | 516,51 | 8,64 | 3,59 | 0,91 | 10,63 | 66,3 |
| X16S_KZN_NHL_H9 | 314 | 314,1 | 0,38 | 314,59 | 6,66 | 3,87 | 0,95 | 20,83 | 39,02 |
| X16S_Limp_EM_FC1 | 80 | 80 | 0,17 | 80,46 | 3,08 | 2,48 | 0,83 | 5,82 | 9,99 |
| X16S_Limp_EM_FC16 | 164 | 164 | 0 | 164 | 4,28 | 3,81 | 0,96 | 22,76 | 20,28 |
| X16S_Limp_EM_FC2 | 239 | 239,07 | 0,29 | 239,69 | 6,13 | 4,16 | 0,97 | 35,86 | 32,75 |
| X16S_Limp_EM_FC21 | 169 | 169 | 0 | 169 | 3,91 | 3,77 | 0,95 | 20,66 | 22,66 |
| X16S_Limp_EM_FC22 | 443 | 443,04 | 0,21 | 443,45 | 10,35 | 3,42 | 0,88 | 8,58 | 68,46 |
| X16S_Limp_EM_FC3 | 345 | 345,14 | 0,49 | 345,48 | 6,09 | 4,31 | 0,97 | 32,17 | 47,45 |
| X16S_Limp_EM_FC4 | 314 | 314,2 | 0,62 | 314,37 | 6,05 | 3,66 | 0,87 | 7,49 | 44,18 |
| X16S_Limp_EM_FC5 | 368 | 368 | 0,03 | 368,28 | 6,82 | 4,54 | 0,98 | 46,81 | 47,8 |
| X16S_Limp_EM_FC6 | 542 | 542 | 0,05 | 542,18 | 9,57 | 5,1 | 0,99 | 78,45 | 84,33 |
| X16S_Limp_EM_FC7 | 61 | 61 | 0 | 61 | 2,14 | 2,81 | 0,91 | 10,97 | 7,03 |
| X16S_Limp_EM_FC8 | 83 | 83 | 0,25 | 83,29 | 3,74 | 2,43 | 0,79 | 4,83 | 11,45 |
| X16S_Limp_FTK_FC11 | 179 | 179 | 0,12 | 179,23 | 5,29 | 3,88 | 0,96 | 22,77 | 24,78 |
| X16S_Limp_FTK_FC12 | 159 | 159 | 0,06 | 159,31 | 5,11 | 3,36 | 0,93 | 14,78 | 18,8 |
| X16S_Limp_FTK_FC13 | 229 | 229 | 0 | 229 | 5,03 | 3,8 | 0,96 | 24,37 | 27,41 |
| X16S_Limp_FTK_FC14 | 187 | 187 | 0,1 | 187,28 | 4,55 | 3,56 | 0,93 | 14,86 | 22,85 |
| X16S_Limp_FTK_FC15 | 260 | 260,27 | 0,66 | 261,24 | 5,63 | 3,81 | 0,93 | 14,89 | 32,3 |
| X16S_Limp_FTK_FC17 | 237 | 237 | 0,07 | 237,21 | 6,17 | 4,18 | 0,98 | 40,47 | 30,47 |
| X16S_Limp_FTK_FC20 | 158 | 158 | 0,1 | 158,29 | 4,49 | 3,02 | 0,83 | 5,78 | 19,68 |
| X16S_Limp_FTK_FC23 | 158 | 158 | 0 | 158 | 4,51 | 3,55 | 0,93 | 15,2 | 20,15 |
| X16S_Limp_FTK_FC24 | 92 | 92 | 0,25 | 92,5 | 2,18 | 3,21 | 0,92 | 12,08 | 11,31 |
| X16S_Limp_FTK_FC27 | 725 | 725,15 | 0,41 | 726,65 | 12,49 | 4,37 | 0,97 | 28,58 | 93,99 |
| X16S_Limp_MM_FC25 | 436 | 436,11 | 0,41 | 436,52 | 6,68 | 5,08 | 0,99 | 67,51 | 66,25 |
| X16S_Limp_MM_FC26 | 117 | 117 | 0,17 | 117,37 | 3,97 | 3,18 | 0,91 | 11,57 | 14,98 |
| X16S_Limp_MM_FC28 | 412 | 412,13 | 0,41 | 412,81 | 8,95 | 3,85 | 0,96 | 25,72 | 45,49 |
| X16S_Limp_MM_FC29 | 813 | 813,52 | 0,85 | 814,8 | 12,65 | 3,65 | 0,89 | 9,12 | 99,59 |
| X16S_Limp_MM_FC30 | 467 | 467,04 | 0,23 | 467,52 | 9,12 | 3,99 | 0,96 | 26,7 | 53,85 |
| X16S_Limp_MM_FC31 | 631 | 631,18 | 0,47 | 632,36 | 10,23 | 4,53 | 0,98 | 46,59 | 71,99 |
| X16S_Limp_MM_FC32 | 755 | 755,11 | 0,36 | 755,79 | 10,83 | 4,33 | 0,96 | 22,25 | 93,03 |
| X16S_Limp_MM_FC33 | 567 | 567,27 | 0,61 | 568,12 | 9,76 | 3,85 | 0,92 | 12,88 | 65,93 |
| X16S_Limp_MM_FC34 | 530 | 530,09 | 0,32 | 530,89 | 9,98 | 4,16 | 0,96 | 23,7 | 64,19 |
| X16S_Limp_MM_FC35 | 1015 | 1015,21 | 0,48 | 1017,95 | 15,46 | 4,85 | 0,98 | 46,29 | 145,38 |

**Supplementary Table 1**: Alpha diversity indices observed in KwaZulu-Natal and Limpopo village chicken fecal microbiome.

| *Sample* | *Abundance* | *Province* | *SampleType* | *Kingdom* | *Phylum* | *Class* | *Order* | *Family* | *Genus* | |  |  |
| --- | --- | --- | --- | --- | --- | --- | --- | --- | --- | --- | --- | --- |
| *16S_KZN_MBL_H12* | 4,49374573 | KwaZulu-Natal | Ethekwini | Bacteria | Proteobacteria | Gammaproteobacteria | Enterobacterales | Enterobacteriaceae | Escherichia-Shigella | |  |  |
| *16S_KZN_NHL_H1* | 4,05587162 | KwaZulu-Natal | Hluhluwe | Bacteria | Firmicutes | Bacilli | Lactobacillales | Lactobacillaceae | Lactobacillus | |  | |
| *16S_Limp_FTK_FC20* | 3,999270267 | Limpopo | Fetakgomo | Bacteria | Bacteroidota | Bacteroidia | Flavobacteriales | Flavobacteriaceae | Flavobacterium | |  | |
| *16S_Limp_EM_FC4* | 3,526716991 | Limpopo | Elias Motsoaledi | Bacteria | Bacteroidota | Bacteroidia | Sphingobacteriales | Sphingobacteriaceae | Pedobacter | |  | |
| *16S_Limp_EM_FC8* | 3,308741476 | Limpopo | Elias Motsoaledi | Bacteria | Proteobacteria | Gammaproteobacteria | Pseudomonadales | Pseudomonadaceae | Pseudomonas | |  | |
| *16S_KZN_MK_H4* | 3,139483226 | KwaZulu-Natal | Mkhambathini | Bacteria | Proteobacteria | Gammaproteobacteria | Enterobacterales | Enterobacteriaceae | Escherichia-Shigella | |  | |
| *16S_KZN_MK_H2* | 3,041883146 | KwaZulu-Natal | Mkhambathini | Bacteria | Proteobacteria | Gammaproteobacteria | Enterobacterales | Enterobacteriaceae | Escherichia-Shigella | |  | |
| *16S_Limp_EM_FC8* | 3,029758215 | Limpopo | Elias Motsoaledi | Bacteria | Firmicutes | Bacilli | Paenibacillales | Paenibacillaceae | Paenibacillus | |  | |
| *16S_Limp_MM_FC29* | 2,986992917 | Limpopo | Mole Mole | Bacteria | Bacteroidota | Bacteroidia | Flavobacteriales | Flavobacteriaceae | Aequorivita | |  | |
| *16S_Limp_EM_FC22* | 2,926160815 | Limpopo | Elias Motsoaledi | Bacteria | Firmicutes | Bacilli | Paenibacillales | Paenibacillaceae | NA |  | |  |
| *16S_Limp_EM_FC1* | 2,771596533 | Limpopo | Elias Motsoaledi | Bacteria | Firmicutes | Bacilli | Paenibacillales | Paenibacillaceae | Paenibacillus | |  | |
| *16S_KZN_NHL_H10* | 2,744280421 | KwaZulu-Natal | Hluhluwe | Bacteria | Proteobacteria | Gammaproteobacteria | Enterobacterales | Enterobacteriaceae | Escherichia-Shigella | |  | |
| *16S_Limp_EM_FC1* | 2,709156731 | Limpopo | Elias Motsoaledi | Bacteria | Firmicutes | Clostridia | Lachnospirales | Lachnospiraceae | NA |  | |  |
| *16S_KZN_MBL_H10* | 2,702760798 | KwaZulu-Natal | Ethekwini | Bacteria | Proteobacteria | Gammaproteobacteria | Enterobacterales | Enterobacteriaceae | Escherichia-Shigella | |  | |
| *16S_KZN_NHL_H8* | 2,591129783 | KwaZulu-Natal | Hluhluwe | Bacteria | Proteobacteria | Gammaproteobacteria | Pseudomonadales | Moraxellaceae | Psychrobacter | |  | |
| *16S_KZN_MBL_H8* | 2,547105736 | KwaZulu-Natal | Ethekwini | Bacteria | Proteobacteria | Gammaproteobacteria | Enterobacterales | Enterobacteriaceae | Escherichia-Shigella | |  | |
| *16S_Limp_MM_FC33* | 2,496042216 | Limpopo | Mole Mole | Bacteria | Bacteroidota | Bacteroidia | Bacteroidales | Dysgonomonadaceae | Fermentimonas | |  | |
| *16S_Limp_FTK_FC15* | 2,248257452 | Limpopo | Fetakgomo | Bacteria | Bacteroidota | Bacteroidia | Sphingobacteriales | Sphingobacteriaceae | Pedobacter | |  | |
| *16S_Limp_FTK_FC24* | 2,133800987 | Limpopo | Fetakgomo | Bacteria | Firmicutes | Bacilli | Lactobacillales | Lactobacillaceae | Lactobacillus | |  | |
| *16S_KZN_NHL_H7* | 2,092375367 | KwaZulu-Natal | Hluhluwe | Bacteria | Proteobacteria | Gammaproteobacteria | Enterobacterales | Enterobacteriaceae | Escherichia-Shigella | |  | |
| *16S_Limp_FTK_FC14* | 2,084732722 | Limpopo | Fetakgomo | Bacteria | Bacteroidota | Bacteroidia | Sphingobacteriales | Sphingobacteriaceae | Pedobacter | |  | |
| *16S_KZN_MBL_H6* | 2,058908718 | KwaZulu-Natal | Ethekwini | Bacteria | Proteobacteria | Gammaproteobacteria | Enterobacterales | Enterobacteriaceae | Escherichia-Shigella | |  | |
| *16S_Limp_FTK_FC23* | 1,875402541 | Limpopo | Fetakgomo | Bacteria | Proteobacteria | Gammaproteobacteria | Pseudomonadales | Pseudomonadaceae | Pseudomonas | |  | |
| *16S_Limp_EM_FC7* | 1,860222109 | Limpopo | Elias Motsoaledi | Bacteria | Firmicutes | Clostridia | Lachnospirales | Lachnospiraceae | Anaerosporobacter | |  | |
| *16S_Limp_MM_FC26* | 1,777861914 | Limpopo | Mole Mole | Bacteria | Proteobacteria | Gammaproteobacteria | Pseudomonadales | Moraxellaceae | Psychrobacter | |  | |
| *16S_Limp_FTK_FC12* | 1,643710033 | Limpopo | Fetakgomo | Bacteria | Proteobacteria | Gammaproteobacteria | Pseudomonadales | Pseudomonadaceae | Pseudomonas | |  | |
| *16S_Limp_MM_FC26* | 1,537983071 | Limpopo | Mole Mole | Bacteria | Bacteroidota | Bacteroidia | Sphingobacteriales | Sphingobacteriaceae | Pedobacter | |  | |
| *16S_KZN_NHL_H9* | 1,431093375 | KwaZulu-Natal | Hluhluwe | Bacteria | Proteobacteria | Gammaproteobacteria | Burkholderiales | Comamonadaceae | Comamonas | |  | |
| *16S_KZN_MK_H1* | 1,41438605 | KwaZulu-Natal | Mkhambathini | Bacteria | Proteobacteria | Gammaproteobacteria | Enterobacterales | Enterobacteriaceae | Escherichia-Shigella | |  | |
| *16S_KZN_NHL_H4* | 1,408634223 | KwaZulu-Natal | Hluhluwe | Bacteria | Firmicutes | Bacilli | Lactobacillales | Lactobacillaceae | Lactobacillus | |  | |
| *16S_Limp_MM_FC32* | 1,388234387 | Limpopo | Mole Mole | Bacteria | Deinococcota | Deinococci | Deinococcales | Trueperaceae | Truepera |  | |  |
| *16S_KZN_MK_H3* | 1,386165544 | KwaZulu-Natal | Mkhambathini | Bacteria | Firmicutes | Bacilli | Lactobacillales | Lactobacillaceae | Lactobacillus | |  | |
| *16S_KZN_MBL_H5* | 1,375725635 | KwaZulu-Natal | Ethekwini | Bacteria | Firmicutes | Bacilli | Lactobacillales | Lactobacillaceae | Lactobacillus | |  | |
| *16S_KZN_MBL_H6* | 1,36978366 | KwaZulu-Natal | Ethekwini | Bacteria | Firmicutes | Bacilli | Bacillales | Planococcaceae | Kurthia |  | |  |
| *16S_Limp_MM_FC26* | 1,366795576 | Limpopo | Mole Mole | Bacteria | Proteobacteria | Gammaproteobacteria | Pseudomonadales | Moraxellaceae | Acinetobacter | |  | |
| *16S_Limp_EM_FC7* | 1,340186144 | Limpopo | Elias Motsoaledi | Bacteria | Firmicutes | Bacilli | Lactobacillales | Lactobacillaceae | Lactobacillus | |  | |
| *16S_Limp_EM_FC21* | 1,330411964 | Limpopo | Elias Motsoaledi | Bacteria | Firmicutes | Bacilli | Bacillales | Bacillaceae | NA |  | |  |
| *16S_KZN_NHL_H3* | 1,325496696 | KwaZulu-Natal | Hluhluwe | Bacteria | Firmicutes | Bacilli | Lactobacillales | Lactobacillaceae | Lactobacillus | |  | |
| *16S_Limp_MM_FC34* | 1,312004269 | Limpopo | Mole Mole | Bacteria | Bacteroidota | Bacteroidia | Bacteroidales | Dysgonomonadaceae | Proteiniphilum | |  | |
| *16S_Limp_MM_FC32* | 1,264541472 | Limpopo | Mole Mole | Bacteria | Proteobacteria | Gammaproteobacteria | Methylococcales | Methylococcaceae | NA |  | |  |
| *16S_Limp_EM_FC1* | 1,263409612 | Limpopo | Elias Motsoaledi | Bacteria | Firmicutes | Clostridia | Oscillospirales | Ruminococcaceae | NA |  | |  |
| *16S_Limp_EM_FC16* | 1,262302552 | Limpopo | Elias Motsoaledi | Bacteria | Firmicutes | Bacilli | Bacillales | Planococcaceae | Sporosarcina | |  | |
| *16S_Limp_FTK_FC11* | 1,262215943 | Limpopo | Fetakgomo | Bacteria | Proteobacteria | Gammaproteobacteria | Enterobacterales | Aeromonadaceae | Oceanisphaera | |  | |
| *16S_KZN_NHL_H2* | 1,259877785 | KwaZulu-Natal | Hluhluwe | Bacteria | Cyanobacteria | Cyanobacteriia | Chloroplast | NA | NA |  | |  |
| *16S_KZN_NHL_H3* | 1,235941754 | KwaZulu-Natal | Hluhluwe | Bacteria | Firmicutes | Bacilli | Mycoplasmatales | Mycoplasmataceae | Candidatus Bacilloplasma | | | |
| *16S_KZN_NHL_H7* | 1,218216319 | KwaZulu-Natal | Hluhluwe | Bacteria | Firmicutes | Bacilli | Lactobacillales | Lactobacillaceae | Lactobacillus | |  | |
| *16S_KZN_NHL_H1* | 1,177829835 | KwaZulu-Natal | Hluhluwe | Bacteria | Firmicutes | Bacilli | Lactobacillales | Lactobacillaceae | Lactobacillus | |  | |
| *16S_Limp_FTK_FC27* | 1,176246832 | Limpopo | Fetakgomo | Bacteria | Proteobacteria | Gammaproteobacteria | Enterobacterales | Enterobacteriaceae | Enterobacter | |  | |
| *16S_KZN_MK_H3* | 1,167336342 | KwaZulu-Natal | Mkhambathini | Bacteria | Proteobacteria | Gammaproteobacteria | Enterobacterales | Enterobacteriaceae | Escherichia-Shigella | |  | |
| *16S_Limp_FTK_FC24* | 1,160820992 | Limpopo | Fetakgomo | Bacteria | Firmicutes | Bacilli | Bacillales | Bacillaceae | NA |  | |  |
| *16S_KZN_MK_H3* | 1,146689745 | KwaZulu-Natal | Mkhambathini | Bacteria | Firmicutes | Bacilli | Lactobacillales | Lactobacillaceae | Lactobacillus | |  | |
| *16S_Limp_FTK_FC12* | 1,108596005 | Limpopo | Fetakgomo | Bacteria | Proteobacteria | Gammaproteobacteria | Pseudomonadales | Pseudomonadaceae | Pseudomonas | |  | |
| *16S_Limp_EM_FC22* | 1,087429219 | Limpopo | Elias Motsoaledi | Bacteria | Actinobacteriota | Actinobacteria | Micrococcales | Cellulomonadaceae | Oerskovia |  | |  |
| *16S_Limp_EM_FC7* | 1,080411169 | Limpopo | Elias Motsoaledi | Bacteria | Actinobacteriota | Actinobacteria | Micrococcales | Micrococcaceae | Arthrobacter | |  | |
| *16S_Limp_FTK_FC11* | 1,069115742 | Limpopo | Fetakgomo | Bacteria | Proteobacteria | Gammaproteobacteria | Cardiobacteriales | Wohlfahrtiimonadaceae | Ignatzschineria | |  | |
| *16S_Limp_EM_FC22* | 1,064552661 | Limpopo | Elias Motsoaledi | Bacteria | Firmicutes | Bacilli | Bacillales | Planococcaceae | Sporosarcina | |  | |
| *16S_Limp_FTK_FC13* | 1,050555231 | Limpopo | Fetakgomo | Bacteria | Firmicutes | Clostridia | Lachnospirales | Lachnospiraceae | Herbinix |  | |  |
| *16S_Limp_MM_FC28* | 1,034170586 | Limpopo | Mole Mole | Bacteria | Bacteroidota | Bacteroidia | Sphingobacteriales | Sphingobacteriaceae | Pedobacter | |  | |
| *16S_Limp_FTK_FC23* | 1,03070047 | Limpopo | Fetakgomo | Bacteria | Bacteroidota | Bacteroidia | Bacteroidales | Bacteroidaceae | Bacteroides | |  | |
| *16S_KZN_NHL_H3* | 1,010313442 | KwaZulu-Natal | Hluhluwe | Bacteria | Firmicutes | Bacilli | Mycoplasmatales | Mycoplasmataceae | Candidatus Bacilloplasma | | | |

**Supplementary Table 2**: Taxonomic table of fecal microbiome observed in KwaZulu-Natal and Limpopo village chicken fecal microbiome.

| MAG | GTDBK Classification |
| --- | --- |
| Bin.002.fastaLimpopo_assembly | p__Proteobacteria;  c__Gammaproteobacteria  o__Burkholderiales  f__Burkholderiaceae  g__Achromobacter  s__Achromobacter spanius |
| Bin.004.fastaLimpopo_assembly | p__Bacteroidota  c__Bacteroidia  o__Flavobacteriales  f__Flavobacteriaceae  g__Gelidibacter  s__ |
| Bin.010.fastaLimpopo_assembly | p__Proteobacteria  c__Gammaproteobacteria  o__Xanthomonadales  f__Xanthomonadaceae  g__Lysobacter s  s__ |
| Bin.014.fastaLimpopo_assembly | p__Bacteroidota  c__Bacteroidia  o__Flavobacteriales  f__Flavobacteriaceae  g__Aequorivita  s__ |
| Bin.015.fastaLimpopo_assembly | p__Firmicutes_A  c__Clostridia  o__Lachnospirales  f__Lachnospiraceae  g__Mediterraneibacter  s__Mediterraneibacter |
| Bin.024.fastaLimpopo_assembly | p__Bacteroidota  c__Bacteroidia  o__Sphingobacteriales  f__Sphingobacteriaceae  g__Arcticibacter  s__ |
| Bin.026.fastaLimpopo_assembly | p__Proteobacteria  c__Gammaproteobacteria  o__Pseudomonadales  f__Pseudomonadaceae  g__Pseudomonas_D  s__ |
| Bin.027.fastaLimpopo_assembly | p__Actinobacteriota  c__Actinomycetia  o__Actinomycetales  f__Bifidobacteriaceae  g__Bifidobacterium  s__Bifidobacterium pseudolongum |
| Bin.028.fastaLimpopo_assembly | p__Firmicutes  c__Bacilli  o__RFN20  f__CAG-826  g__  s__ |
| Bin.030.fastaLimpopo_assembly | p__Bacteroidota  c__Bacteroidia  o__Sphingobacteriales  f__Sphingobacteriaceae  g__  s__ |
| Bin.031.fastaLimpopo_assembly | p__Bacteroidota  c__Bacteroidia  o__Flavobacteriales  f__Flavobacteriaceae  g__Aequorivita  s__ |
| Bin.032.fastaLimpopo_assembly | p__Proteobacteria  c__Gammaproteobacteria  o__Pseudomonadales  f__Pseudohongiellaceae  g__  s__ |
| Bin.034.fastaLimpopo_assembly | p__Firmicutes  c__Bacilli  o__Lactobacillales  f__Lactobacillaceae  g__Lactobacillus  s__Lactobacillus crispatus |
| Bin.036.fastaLimpopo_assembly | p__Actinobacteriota  c__Actinomycetia  o__Actinomycetales  f__Cellulomonadaceae  g__Sanguibacter  s__Sanguibacter suarezii |
| Bin.039.fastaLimpopo_assembly | p__Actinobacteriota  c__Actinomycetia  o__Mycobacteriales  f__Mycobacteriaceae  g__Corynebacterium  s__Corynebacterium variabile |
| Bin.040.fastaLimpopo_assembly | p__Bacteroidota  c__Bacteroidia  o__Bacteroidales  f__UBA932  g__CAG-831  s__ |
| Bin.042.fastaLimpopo_assembly | p__Proteobacteria  c__Alphaproteobacteria  o__Rhodobacterales  f__Rhodobacteraceae  g__Wagnerdoeblera  s__ |
| Bin.044.fastaLimpopo_assembly | p__Actinobacteriota  c__Actinomycetia  o__Actinomycetales  f__Microbacteriaceae  g__Leucobacter  s__ |
| Bin.046.fastaLimpopo_assembly | p__Proteobacteria  c__Gammaproteobacteria  o__Pseudomonadales  f__Moraxellaceae  g__Alkanindiges  s__ |
| Bin.047.fastaLimpopo_assembly | p__Proteobacteria  c__Alphaproteobacteria  o__Sphingomonadales  f__Sphingomonadaceae  g__Parasphingorhabdus  s__ |
| Bin.048.fastaLimpopo_assembly | p__Spirochaetota  c__Spirochaetia  o__Sphaerochaetales  f__Sphaerochaetaceae  g__  s__ |
| Bin.049.fastaLimpopo_assembly | p__Actinobacteriota  c__Thermoleophilia  o__Solirubrobacterales  f__Solirubrobacteraceae  g__SYBY01  s__ |
| Bin.050.fastaLimpopo_assembly | p__Proteobacteria  c__Alphaproteobacteria  o__Rhodobacterales  f__Rhodobacteraceae  g__Roseovarius  s__ |
| Bin.052.fastaLimpopo_assembly | p__Actinobacteriota  c__Actinomycetia  o__Mycobacteriales  f__Mycobacteriaceae  g__Corynebacterium  s__Corynebacterium glutamicum |
| Bin.053.fastaLimpopo_assembly | p__Spirochaetota  c__Spirochaetia  o__Treponematales  f__Treponemataceae  g__  s__ |
| Bin.054.fastaLimpopo_assembly | p__Verrucomicrobiota  c__Verrucomicrobiae  o__Verrucomicrobiales  f__Akkermansiaceae  g__UBA1315  s__ |
| Bin.055.fastaLimpopo_assembly | p__Proteobacteria  c__Gammaproteobacteria  o__Pseudomonadales  f__Cellvibrionaceae  g__Cellvibrio  s__ |
| Bin.057.fastaLimpopo_assembly | p__Bacteroidota  c__Bacteroidia  o__Cytophagales  f__Hymenobacteraceae  g__Botryobacter  s__ |
| Bin.058.fastaLimpopo_assembly | p__Bacteroidota  c__Bacteroidia  o__Bacteroidales  f__Dysgonomonadaceae  g__UBA2632  s__UBA2632 sp002359825 |
| Bin.059.fastaLimpopo_assembly | p__Proteobacteria  c__Gammaproteobacteria  o__Burkholderiales  f__Burkholderiaceae  g__Pusillimonas  s__ |
| Bin.060.fastaLimpopo_assembly | p__Firmicutes_A  c__Clostridia  o__Lachnospirales  f__Lachnospiraceae  g__Anaerobium  s__ |
| Bin.061.fastaLimpopo_assembly | p__Firmicutes  c__Bacilli  o__RFN20  f__CAG-826  g__UBA4855  s__UBA4855 sp900540365 |
| Bin.063.fastaLimpopo_assembly | p__Proteobacteria  c__Alphaproteobacteria  o__Rhizobiales  f__Xanthobacteraceae  g__Bradyrhizobium  s__Bradyrhizobium sp000015165 |
| Bin.068.fastaLimpopo_assembly | p__Actinobacteriota  c__Coriobacteriia  o__Coriobacteriales  f__Coriobacteriaceae  g__Collinsella  s__ |
| Bin.069.fastaLimpopo_assembly | p__Bacteroidota  c__Bacteroidia  o__Flavobacteriales  f__Cryomorphaceae  g__Cryomorpha  s__ |
| Bin.070.fastaLimpopo_assembly | p__Spirochaetota  c__Spirochaetia  o__Sphaerochaetales  f__Sphaerochaetaceae  g__Spiro-01  s__ |
| Bin.071.fastaLimpopo_assembly | p__Spirochaetota  c__Spirochaetia  o__Sphaerochaetales  f__Sphaerochaetaceae  g__Spiro-01  s__ |
| Bin.072.fastaLimpopo_assembly | p__Gemmatimonadota  c__Gemmatimonadetes  o__Longimicrobiales  f__RSA9  g__  s__ |
| Bin.075.fastaLimpopo_assembly | p__Firmicutes  c__Bacilli  o__Lactobacillales  f__Carnobacteriaceae  g__  s__ |
| Bin.081.fastaLimpopo_assembly | p__Bacteroidota  c__Bacteroidia  o__Cytophagales  f__Cyclobacteriaceae  g__  s__ |
| Bin.084.fastaLimpopo_assembly | p__Proteobacteria  c__Gammaproteobacteria  o__Burkholderiales  f__Burkholderiaceae  g__Paenalcaligenes  s__ |
| Bin.085.fastaLimpopo_assembly | p__Bacteroidota  c__Bacteroidia  o__Bacteroidales  f__Dysgonomonadaceae  g__UBA2632  s__ |
| Bin.086.fastaLimpopo_assembly | p__Bacteroidota  c__Bacteroidia  o__Sphingobacteriales  f__Sphingobacteriaceae  g__Daejeonella  s__ |
| Bin.087.fastaLimpopo_assembly | p__Proteobacteria  c__Gammaproteobacteria  o__Burkholderiales  f__Burkholderiaceae  g__Parapusillimonas  s__ |
| Bin.088.fastaLimpopo_assembly | p__Proteobacteria  c__Gammaproteobacteria  o__Pseudomonadales  f__Oleiphilaceae  g__  s__ |
| Bin.089.fastaLimpopo_assembly | p__Spirochaetota  c__Spirochaetia  o__Sphaerochaetales  f__Sphaerochaetaceae  g__  s__ |
| Bin.092.fastaLimpopo_assembly | p__Proteobacteria  c__Gammaproteobacteria  o__Pseudomonadales  f__Pseudomonadaceae  g__Pseudomonas_C  s__Pseudomonas_C sp012719655 |
| Bin.093.fastaLimpopo_assembly | p__Actinobacteriota  c__Actinomycetia  o__Propionibacteriales  f__Nocardioidaceae  g__Aeromicrobium  s__Aeromicrobium taml... |
| Bin.096.fastaLimpopo_assembly | p__Proteobacteria  c__Alphaproteobacteria  o__Rhizobiales  f__Devosiaceae  g__Devosia  s__Devosia elaeis |
| Bin.097.fastaLimpopo_assembly | p__Proteobacteria  c__Gammaproteobacteria  o__Burkholderiales  f__Burkholderiaceae  g__Alcaligenes  s__Alcaligenes faecalis |
| Bin.099.fastaLimpopo_assembly | p__Proteobacteria  c__Gammaproteobacteria  o__Burkholderiales  f__Burkholderiaceae  g__JACCEO01  s__ |
| Bin.100.fastaLimpopo_assembly | p__Proteobacteria  c__Gammaproteobacteria  o__Pseudomonadales  f__Cellvibrionaceae  g__Marinimicrobium  s__ |
| Bin.103.fastaLimpopo_assembly | p__Proteobacteria  c__Gammaproteobacteria  o__Burkholderiales  f__Burkholderiaceae  g__Pusillimonas  s__ |
| Bin.104.fastaLimpopo_assembly | p__Actinobacteriota  c__Actinomycetia  o__Propionibacteriales  f__Nocardioidaceae  g__Aeromicrobium  s__ |
| Bin.105.fastaLimpopo_assembly | p__Proteobacteria  c__Gammaproteobacteria  o__Enterobacterales  f__Enterobacteriaceae  g__Escherichia  s__Escherichia flexneri |
| Bin.106.fastaLimpopo_assembly | p__Proteobacteria  c__Gammaproteobacteria  o__Burkholderiales  f__Burkholderiaceae  g__Alcaligenes  s__ |
| Bin.108.fastaLimpopo_assembly | p__Proteobacteria  c__Gammaproteobacteria  o__Nitrococcales  f__AK92  g__JAAYIK01  s__ |
| Bin.109.fastaLimpopo_assembly | p__Bacteroidota  c__Bacteroidia  o__Sphingobacteriales  f__Sphingobacteriaceae  g__Albibacterium  s__ |
| Bin.110.fastaLimpopo_assembly | p__Actinobacteriota  c__Thermoleophilia  o__Solirubrobacterales  f__Solirubrobacteraceae  g__  s__ |
| Bin.113.fastaLimpopo_assembly | p__Verrucomicrobiota  c__Verrucomicrobiae  o__Opitutales  f__UBA953  g__W0P29-029  s__ |
| Bin.118.fastaLimpopo_assembly | p__Firmicutes  c__Bacilli  o__Lactobacillales  f__Lactobacillaceae  g__Ligilactobacillus  s__Ligilactobacillus agilis |
| Bin.119.fastaLimpopo_assembly | p__Proteobacteria  c__Alphaproteobacteria  o__Rhodobacterales  f__Rhodobacteraceae  g__Paracoccus  s__ |
| Bin.121.fastaLimpopo_assembly | p__Actinobacteriota  c__Actinomycetia  o__Mycobacteriales  f__Mycobacteriaceae  g__Corynebacterium  s__Corynebacterium xerosis |
| Bin.122.fastaLimpopo_assembly | p__Firmicutes_A  c__Clostridia  o__Tissierellales  f__Tissierellaceae  g__  s__ |
| Bin.124.fastaLimpopo_assembly | p__Proteobacteria  c__Gammaproteobacteria  o__Burkholderiales  f__Burkholderiaceae  g__Paucimonas  s__ |
| Bin.125.fastaLimpopo_assembly | p__Firmicutes_A  c__Clostridia_A  o__Christensenellales  f__DTU072  g__  s__ |
| Bin.126.fastaLimpopo_assembly | p__Actinobacteriota  c__Actinomycetia  o__Streptosporangiales  f__Streptosporangiaceae  g__Actinorugispora  s__ |
| Bin.130.fastaLimpopo_assembly | p__Bacteroidota  c__Bacteroidia  o__Bacteroidales  f__UBA932  g__UBA3382  s__ |
| Bin.131.fastaLimpopo_assembly | p__Firmicutes_A  c__Clostridia  o__Oscillospirales  f__Ruminococcaceae  g__JAAYSD01  s__ |
| Bin.133.fastaLimpopo_assembly | p__Actinobacteriota  c__Actinomycetia  o__Mycobacteriales  f__Mycobacteriaceae  g__Rhodococcus  s__Rhodococcus sp012844225 |
| Bin.134.fastaLimpopo_assembly | p__Proteobacteria  c__Gammaproteobacteria  o__Burkholderiales  f__Burkholderiaceae  g__  s__ |
| Bin.137.fastaLimpopo_assembly | p__Bacteroidota  c__Bacteroidia  o__Flavobacteriales  f__Weeksellaceae  g__Planobacterium  s__Planobacterium lacus |
| Bin.141.fastaLimpopo_assembly | p__Firmicutes  c__Bacilli  o__Lactobacillales  f__Enterococcaceae  g__Enterococcus_B  s__Enterococcus_B faecium |
| Bin.143.fastaLimpopo_assembly | p__Proteobacteria  c__Gammaproteobacteria  o__Burkholderiales  f__Burkholderiaceae  g__JACCEO01  s__ |
| Bin.145.fastaLimpopo_assembly | p__Bacteroidota  c__Bacteroidia  o__Bacteroidales  f__UBA932  g__RC9  s__ |
| Bin.146.fastaLimpopo_assembly | p__Proteobacteria  c__Gammaproteobacteria  o__Burkholderiales  f__Burkholderiaceae  g__  s__ |
| Bin.147.fastaLimpopo_assembly | p__Proteobacteria  c__Alphaproteobacteria  o__Rhizobiales  f__Devosiaceae  g__Pelagibacterium  s__Pelagibacterium sp... |
| Bin.149.fastaLimpopo_assembly | p__Firmicutes  c__Bacilli  o__Paenibacillales  f__Paenibacillaceae  g__  s__ |
| Bin.150.fastaLimpopo_assembly | p__Bacteroidota  c__Bacteroidia  o__Sphingobacteriales  f__Sphingobacteriaceae  g__  s__ |
| Bin.152.fastaLimpopo_assembly | p__Bacteroidota  c__Bacteroidia  o__Bacteroidales  f__Dysgonomonadaceae  g__Fermentimonas  s__ |
| Bin.153.fastaLimpopo_assembly | p__Planctomycetota  c__Planctomycetes  o__Pirellulales  f__Pirellulaceae  g__UBA11363  s__ |
| Bin.154.fastaLimpopo_assembly | p__Proteobacteria  c__Gammaproteobacteria  o__Pseudomonadales  f__Moraxellaceae  g__Acinetobacter  s__Acinetobacter pseudolwoffii |
| Bin.157.fastaLimpopo_assembly | p__Synergistota  c__Synergistia  o__Synergistales  f__Synergistaceae  g__An23  s__An23 sp900544635 |
| Bin.160.fastaLimpopo_assembly | p__Firmicutes  c__Bacilli  o__Lactobacillales  f__Enterococcaceae  g__Enterococcus_I  s__Enterococcus_I aquimarinus |
| Bin.161.fastaLimpopo_assembly | p__Firmicutes  c__Bacilli  o__Lactobacillales  f__Lactobacillaceae  g__Limosilactobacillus  s__Limosilactobacillus ingluviei |

**Supplementary Table 3**: GTDBK classification of Limpopo province village chicken fecal microbiome bins

| MAG | GTDBK Classification |
| --- | --- |
| Bin.001.fastaKZN_IDBA_assembly | d__Bacteria;  p__Bacteroidota;  c__Bacteroidia;  o__Flavobacteriales;  f__Flavobacteriaceae;  g__Gelidibacter;  s__ |
| Bin.006.fastaKZN_IDBA_assembly | d__Bacteria;  p__Proteobacteria;  c__Gammaproteobacteria;  o__Pseudomonadales;  f__Moraxellaceae;  g__Psychrobacter;  s__ |
| Bin.008.fastaKZN_IDBA_assembly | d__Bacteria;  p__Campylobacterota;  c__Campylobacteria;  o__Campylobacterales;  f__Helicobacteraceae;  g__Helicobacter_H;  s__ |
| Bin.009.fastaKZN_IDBA_assembly | d__Bacteria;  p__Firmicutes;  c__Bacilli;  o__Mycoplasmatales;  f__UBA3375;  g__UBA3375;  s__UBA3375 sp002359095 |
| Bin.010.fastaKZN_IDBA_assembly | d__Bacteria;  p__Actinobacteriota;  c__Actinomycetia;  o__Actinomycetales;  f__Microbacteriaceae;  g__Leucobacter;  s__ |
| Bin.011.fastaKZN_IDBA_assembly | d__Bacteria;  p__Actinobacteriota;  c__Actinomycetia;  o__Actinomycetales;  f__Brevibacteriaceae;  g__Brevibacterium;  s__Brevibacterium yomogidense |
| Bin.012.fastaKZN_IDBA_assembly | d__Bacteria;  p__Proteobacteria;  c__Gammaproteobacteria;  o__Burkholderiales;  f__Burkholderiaceae;  g__Comamonas;  s__ |
| Bin.014.fastaKZN_IDBA_assembly | d__Bacteria;  p__Proteobacteria;  c__Gammaproteobacteria;  o__Pseudomonadales;  f__Pseudomonadaceae;  g__Pseudomonas_D;  s__ |
| Bin.015.fastaKZN_IDBA_assembly | d__Bacteria;  p__Bacteroidota;  c__Bacteroidia;  o__Sphingobacteriales;  f__Sphingobacteriaceae;  g__Pelobium;  s__Pelobium manganitolerans |
| Bin.023.fastaKZN_IDBA_assembly | d__Bacteria;  p__Firmicutes_C;  c__Negativicutes;  o__Veillonellales;  f__Veillonellaceae;  g__Veillonella_A;  s__Veillonella_A magna |
| Bin.024.fastaKZN_IDBA_assembly | d__Bacteria;  p__Bacteroidota;  c__Bacteroidia;  o__Flavobacteriales;  f__Crocinitomicaceae;  g__40-80;  s__ |
| Bin.027.fastaKZN_IDBA_assembly | d__Bacteria;  p__Bacteroidota;  c__Bacteroidia;  o__Chitinophagales;  f__Chitinophagaceae;  g__;  s__ |
| Bin.029.fastaKZN_IDBA_assembly | d__Bacteria;  p__Bacteroidota;  c__Bacteroidia;  o__Sphingobacteriales;  f__Sphingobacteriaceae;  g__Sphingobacterium;  s__ |
| Bin.033.fastaKZN_IDBA_assembly | d__Bacteria;  p__Bacteroidota;  c__Bacteroidia;  o__Sphingobacteriales;  f__Sphingobacteriaceae;  g__Arcticibacter;  s__ |
| Bin.035.fastaKZN_IDBA_assembly | d__Bacteria;  p__Proteobacteria;  c__Gammaproteobacteria;  o__Enterobacterales;  f__Aeromonadaceae;  g__Oceanisphaera;  s__ |
| Bin.039.fastaKZN_IDBA_assembly | d__Bacteria;  p__Actinobacteriota;  c__Actinomycetia;  o__Actinomycetales;  f__Bifidobacteriaceae;  g__Bifidobacterium;  s__ |
| Bin.040.fastaKZN_IDBA_assembly | d__Bacteria;  p__Bacteroidota;  c__Bacteroidia;  o__Sphingobacteriales;  f__Sphingobacteriaceae;  g__Sphingobacterium;  s__ |
| Bin.042.fastaKZN_IDBA_assembly | d__Bacteria;  p__Proteobacteria;  c__Gammaproteobacteria;  o__Burkholderiales;  f__Burkholderiaceae;  g__Sutterella;  s__ |
| Bin.045.fastaKZN_IDBA_assembly | d__Bacteria;  p__Firmicutes;  c__Bacilli;  o__Lactobacillales;  f__Enterococcaceae;  g__Enterococcus;  s__Enterococcus faecalis |
| Bin.046.fastaKZN_IDBA_assembly | d__Bacteria;  p__Bacteroidota;  c__Bacteroidia;  o__Flavobacteriales;  f__Flavobacteriaceae;  g__Aequorivita;  s__ |
| Bin.048.fastaKZN_IDBA_assembly | d__Bacteria;  p__Actinobacteriota;  c__Actinomycetia;  o__Actinomycetales;  f__Cellulomonadaceae;  g__;  s__ |
| Bin.049.fastaKZN_IDBA_assembly | d__Bacteria;  p__Bacteroidota;  c__Bacteroidia;  o__Chitinophagales;  f__Chitinophagaceae;  g__Palsa-955;  s__ |
| Bin.051.fastaKZN_IDBA_assembly | d__Bacteria;  p__Proteobacteria;  c__Alphaproteobacteria;  o__Micavibrionales;  f__GCA-2720935;  g__;  s__ |
| Bin.052.fastaKZN_IDBA_assembly | d__Bacteria;  p__Firmicutes_A;  c__Clostridia;  o__Lachnospirales;  f__Lachnospiraceae;  g__Ruminococcus_B;  s__ |
| Bin.056.fastaKZN_IDBA_assembly | d__Bacteria;  p__Firmicutes;  c__Bacilli;  o__Erysipelotrichales;  f__Erysipelatoclostridiaceae;  g__UBA3379;  s__UBA3379 sp002359025 |
| Bin.057.fastaKZN_IDBA_assembly | d__Bacteria;  p__Bacteroidota;  c__Bacteroidia;  o__Sphingobacteriales;  f__Sphingobacteriaceae;  g__Sphingobacterium;  s__Sphingobacterium sp000938735 |
| Bin.058.fastaKZN_IDBA_assembly | d__Bacteria;  p__Proteobacteria;  c__Gammaproteobacteria;  o__Enterobacterales;  f__Pasteurellaceae;  g__Gallibacterium;  s__Gallibacterium anatis |
| Bin.059.fastaKZN_IDBA_assembly | d__Bacteria;  p__Proteobacteria;  c__Gammaproteobacteria;  o__Enterobacterales;  f__Aeromonadaceae;  g__Oceanisphaera;  s__Oceanisphaera sp012518835 |
| Bin.061.fastaKZN_IDBA_assembly | d__Bacteria;  p__Bacteroidota;  c__Bacteroidia;  o__Flavobacteriales;  f__Weeksellaceae;  g__Moheibacter;  s__ |
| Bin.063.fastaKZN_IDBA_assembly | d__Bacteria;  p__Actinobacteriota;  c__Actinomycetia;  o__Mycobacteriales;  f__Mycobacteriaceae;  g__Rhodococcus;  s__ |

**Supplementary Table 4**: GTDBK classification of KwaZulu-Natal province village chicken fecal microbiome bins
